# Anti-Tumor Strategies by Harnessing the Phagocytosis of Macrophages

**DOI:** 10.3390/cancers15102717

**Published:** 2023-05-11

**Authors:** Si-Yuan Li, Yong-Lin Guo, Jia-Wen Tian, He-Jing Zhang, Rui-Fang Li, Ping Gong, Zi-Li Yu

**Affiliations:** 1 The State Key Laboratory Breeding Base of Basic Science of Stomatology (Hubei-MOST) & Key Laboratory of Oral Biomedicine Ministry of Education, School and Hospital of Stomatology, Wuhan University, Wuhan 430079, China; 2 Department of Anesthesiology, School and Hospital of Stomatology, Wuhan University, Wuhan 430079, China; 3 Department of Oral and Maxillofacial Surgery, School and Hospital of Stomatology, Wuhan University, Wuhan 430079, China

**Keywords:** macrophages, phagocytic signals, immunotherapy, CAR-macrophage, nanomedicine

## Abstract

**Simple Summary:**

Macrophages are the “big eaters” of the immune system who are in charge of engulfing undesirable substances. Macrophages are vital for the human body as they are instrumental in developing organisms, regulating immune responses, and maintaining a relatively stable internal environment. When the phagocytic capacity of macrophages is impaired, the body is prone to disease. In the context of tumors, tumor cells have their ways to escape from macrophage-mediated phagocytosis. They masquerade as healthy cells by expressing “don’t eat me” signals to fool macrophages and turn the initially anti-tumoral macrophages against the human body, which results in reduced macrophage-mediated phagocytosis. Hence, promoting the phagocytosis of macrophages is an important approach to improving the efficacy of anti-tumor treatment. In this review, we introduced the underlying mechanisms of macrophage-mediated phagocytosis and reviewed the recent progress in the area of application strategies on the basis of the phagocytosis mechanism.

**Abstract:**

Macrophages are essential for the human body in both physiological and pathological conditions, engulfing undesirable substances and participating in several processes, such as organism growth, immune regulation, and maintenance of homeostasis. Macrophages play an important role in anti-bacterial and anti-tumoral responses. Aberrance in the phagocytosis of macrophages may lead to the development of several diseases, including tumors. Tumor cells can evade the phagocytosis of macrophages, and “educate” macrophages to become pro-tumoral, resulting in the reduced phagocytosis of macrophages. Hence, harnessing the phagocytosis of macrophages is an important approach to bolster the efficacy of anti-tumor treatment. In this review, we elucidated the underlying phagocytosis mechanisms, such as the equilibrium among phagocytic signals, receptors and their respective signaling pathways, macrophage activation, as well as mitochondrial fission. We also reviewed the recent progress in the area of application strategies on the basis of the phagocytosis mechanism, including strategies targeting the phagocytic signals, antibody-dependent cellular phagocytosis (ADCP), and macrophage activators. We also covered recent studies of Chimeric Antigen Receptor Macrophage (CAR-M)-based anti-tumor therapy. Furthermore, we summarized the shortcomings and future applications of each strategy and look into their prospects with the hope of providing future research directions for developing the application of macrophage phagocytosis-promoting therapy.

## 1. Introduction

Macrophages are crucial phagocytes of the immune system in both physiological and pathological conditions as they assume the role of the forefront of innate immune defense against invaders [1], silently engulfing foreign bodies, waste products, aging cells, and tumor cells [2,3,4]. Characterized by avid phagocytosis, macrophages are referred to as “the big eaters” in Greek by Ilya (Elie) Metchnikoff, the father of cellular immunology [5]. The process of the rapid and efficient elimination of undesirable cells by macrophages is meaningful for several important functions, including organism growth, immunoregulation, and tissue homeostasis maintenance [6,7]. Macrophages strategically dwell in all tissue and engage in various stages of pathology [8] and play a pivotal part in anti-bacterial and anti-tumoral responses by recognizing specific stimulus ligands, engulfing diseased cells, and digesting internalized cargos.

According to mounting evidence, defects in macrophage phagocytosis are associated with the development and progression of several diseases [6]. If not eliminated as they should be, uncleared cells can be prone to secondary necrosis, release toxic intracellular components to microenvironments, and cause harmful effects that potentially stimulate inflammation, types of tumors, neurodegenerative disorders, kidney problems, asthma, and so forth [6,7]. In the scenario of tumors, tumor cells can circumvent macrophage phagocytosis in various modes, including the overexpression of “don’t eat me” signals and mucins, as well as the “educational” nature of tumor microenvironment (TME) that shifts macrophages from anti-tumoral to pro-tumoral status [9], resulting in the reduced phagocytic ability of macrophages and a massive increase in tumor-associated macrophages (TAMs) in tumor patients [10,11]. Additionally, TAMs abundant in TME are frequently associated with bad prognoses [12,13].

Consequently, harnessing the phagocytosis of macrophages is an important approach to bolstering the efficacy of anti-tumor treatment. In our review, we emphatically introduced the underlying phagocytosis mechanism, such as the equilibrium among phagocytic signals, receptors and their respective signaling pathways, macrophage activation, as well as mitochondrial fission, which can directly augment macrophage phagocytosis. Our review also covered the recent advancements in anti-tumor strategies for enhancing macrophage phagocytosis including strategies targeting the phagocytic signals, antibody-dependent cellular phagocytosis (ADCP), and macrophage activators. Furthermore, our review highlights Chimeric Antigen Receptor Macrophage (CAR-M) as the upcoming generation acting as the link between innate and adaptive immunity, thereby promoting effective tumoricidal immune responses.

## 2. Mechanisms of Phagocytosis

Phagocytosis is secondary to particle ligands binding to the phagocytic receptors, such as Fc gamma receptor (FcγR) and complement receptor (CR), on the phagocyte cell surface and this event requires actin assembly, pseudopod extension, and phagosome closure [14]. For phagocytosis events, extracellular players include the “find me” signal, “eat me” signal, “don’t eat me” signal and their receptors, as well as phagocytic receptors, while intracellular players include actin, pseudopod, and phagosome. Both enhancing macrophage phagocytic capacity and regulating phagocytic signals can promote macrophage-mediated phagocytosis. 

**Figure 1:**
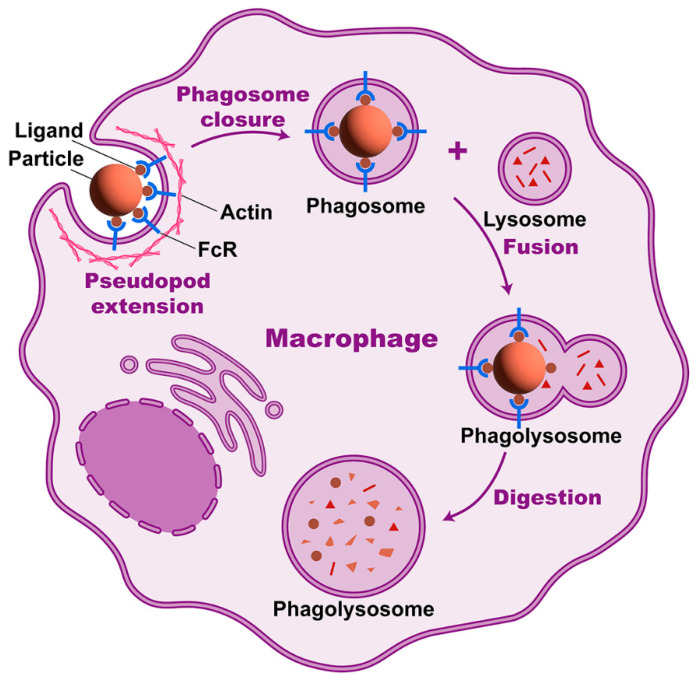
Phagocytosis process of macrophages. After particle ligands bind to phagocytic receptors, macrophages engulf the particle in a process involving actin assembly, pseudopod extension, and phagosome closure. The phagosome fuses with the lysosome and becomes a phagolysosome, where particle digestion takes place.

Taking FcγR-mediated phagocytosis as an example, IgG coated on the particle binds to FcγR and triggers receptor aggregation, inducing actin aggregation at the site of ingestion to produce the protrusive force for pseudopod extension [14]. To initiate the reshaping involved in pseudopodia extension, it is necessary to partially disassemble the F-actin networks, which support quiescent macrophages’ cytoskeleton. The disassembly also promotes the lateral diffusion and free aggregation of receptors [15]. The next two steps are phagosome closure and actin depolymerization from the phagocytic cup, which are accompanied by the full extension of pseudopodia around the phagocytic targets [15]. Additionally, the depolymerization of actin filaments from the advanced phagocytic cup may promote membrane curvature [15]. Some other intracellular players can trigger the actinomyosin contractility involved in phagosome closure [14]. Subsequently, with the coordinated interaction of the actin and tubulin-based cytoskeletons, the phagosome undergoes maturation by a series of fusion and fission events [16] (Figure 1).

### 2.1. Phagocytic Signals

**Figure 2:**
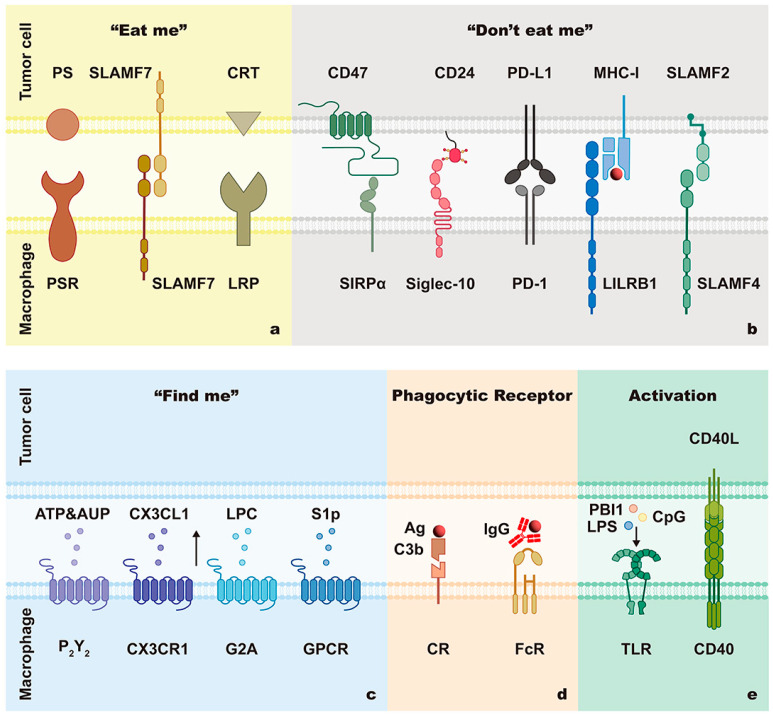
Interactions between macrophages and tumor cells in FcγR-mediated phagocytosis. The phagocytosis of macrophages is related to phagocytic signals, phagocytic receptors, and macrophage activators. Phagocytic signals, including “eat me” signals (**a**), “don’t eat me” signals (**b**), and “find me” signals (**c**), function as phagocytosis switches. Macrophages recognize phagocyte-specific antigens and ligands through various phagocytic receptors (**d**). The capability of macrophage-mediated phagocytosis is influenced by macrophage activation (**e**).

Phagocytic signals are not signals that transmit information, but a special class of substances that act as phagocytosis switches, including “find me”, “eat me”, and “don’t eat me” signals (Figure 2 and Table 1). These signals dictate whether the engulfment occurs. “Find me” signals publicize the presence of apoptotic cells and recruit phagocytes. “Eat me” signals are exposed to abnormal cells and promote phagocytosis. On the contrary, “don’t eat me” signals put the brakes on phagocytosis. It is generally believed that the balance between “eat me” and “don’t eat me” signals is essential to maintain normal phagocytosis in vivo [17]. While “eat me” signals are not usually expressed in living human cells, “don’t eat me” signals are expressed commonly among various cell types, including tumor cells [17,18]. Of particular note is that the secretion of “find me” signals, exposure of “eat me” signals, and deficiency of “don’t eat me” signals are known as the three critical factors of apoptotic cell clearance [19]. 

#### 2.1.1. “Find Me” Signals

Common “find me” signals include nucleotides (ATP, UTP), CX3CL1, and LPC. Apoptotic cells secrete “find me” signals to indicate their location and recruit macrophages. Macrophages migrate toward the vicinity of apoptotic cells by recognizing the “find me” signal gradient and initiate phagocytosis [38]. However, due to the “educational” nature of TME, macrophages attracted to TME by “find me” signals may shift from anti-tumoral to pro-tumoral status. Although “find me” signals are poorly explored in tumor therapy, there is a wide application foreground in this area.

#### 2.1.2. “Don’t Eat Me” Signals

##### CD47/SIRPα

Tumor cells can evade macrophage-mediated phagocytosis by expressing various types of “don’t eat me” signals that interact with macrophage receptors. CD47, a receptor of signal regulatory protein α (SIRPα), is one of the “don’t eat me” signals expressed on healthy cells and frequently over-expressed on tumor cells. SIRPα contains immunoreceptor tyrosine-based inhibition motifs (ITIMs) that are phosphorylated and attract inhibitory molecules, such as Src homology 2 (SH2) domain-containing protein tyrosine phosphatase (SHP)-1 and SHP-2. When CD47 binds to SIRPα, SIRPα is coupled to the phosphatases, which stops macrophage activation [39]. A study showed that the unsustainable effectiveness of anti-angiogenic therapy is due to its potential to promote CD47 expression, imparting tolerance to anti-angiogenic treatment for non-small cell lung cancer [40]. CD47/SIRPα blockade elicits anti-tumor activity by facilitating macrophage phagocytosis, which is amplified by CD40 signaling [41].

##### CD24/Siglec-10

Numerous tumors overexpress CD24 and TAMs overexpress sialic-acid-binding Ig-like lectin 10 (Siglec-10). In the TME, CD24 mediates immune escape through its interaction with Siglec-10. It has been shown that treatment with monoclonal antibodies (mAbs) could prevent the connection of CD24 and Siglec-10, effectively harnessing the phagocytosis of the tumor [34]. 

##### MHC-I/LILRB1

β2-microglobulin (β2M), a component of major histocompatibility complex I (MHC-I), is expressed by tumor cells and protects the tumor from phagocytosis. MHC-I expression is increased in macrophages, including TAMs. Leukocyte immunoglobulin-like receptor B1 (LILRB1) is the receptor of MHC-I. Disrupting either MHC-I or LILRB1 can facilitate phagocytosis, suggesting that the MHC-I/LILRB1 axis plays a vital role in inhibiting macrophage phagocytosis, which shows potential to be a possible marker for therapeutic response to CD47-targeted agents and target of anti-tumor therapy [37].

##### SLAMF3/SLAMF3 and SLAMF2/SLAMF4

In contrast to widely-expressed CD47, SLAM family receptors (SFRs) are only expressed on hematopoietic cells. Specific SFRs, especially SLAMF3 and SLAMF4, function as receptors of “don’t eat me” signals on macrophages. SLAMF3 recognizes itself as self-ligand, while SLAMF4 (also known as 2B4) binds to SLAMF2 (also known as CD48). These receptors can inhibit “eat me” signals with SH2-domain-containing phosphatases (SHPs). SFRs combined with CD47 suppress LRP1 signaling to inhibit macrophage phagocytosis which is crucial to hematopoietic homeostasis. Of note, SFRs are independent of CD47 in this process [33].

#### 2.1.3. “Eat Me” Signals

As effective pro-phagocytic signals, “eat me” signals are up-regulated on the tumor cell surface to fight tumors, which has achieved many successes [42,43]. It is worth noting that “eat me” and “don’t eat me” signals can be concealed by coating layers on the cell surface in both steric and electrostatic ways. The elimination of these physical barriers using enzymatic means markedly improves the efficiency of phagocytosis. Especially, the elimination of mucins, which are overexpressed in tumor cells, promotes phagocytosis. These findings demonstrate the prospect of the physical barriers to regulating phagocytosis in anti-tumor therapy [11].

##### PS/PSR

Phosphatidylserine (PS) is the most robust “eat me” signal of apoptotic cells. PtdSer is recognized by receptors on macrophages, including CD300b, brain-specific angiogenesis inhibitor 1 (BAI1), T cell immunoglobulin, mucin domain-containing molecule 4 (TIM4), and stabilin 2. PtdSer is also recognized by soluble, bifunctional bridging proteins, such as GAS6 or protein S (PROS1) and milk fat globule-EGF factor 8 (MFGE8) [44]. Utsugi et al. demonstrated that tumor cells also overexpressed PS [45]. However, unlike apoptotic cells, PS in the outer membrane of tumor cells mainly comes into contact with receptors on immune cells to inhibit anti-tumor response, including dendritic cell and cytotoxic T cell inhibition, as well as inhibitory cytokine secretion promotion [45,46]. For example, the TYRO3, AXL, and MERTK family of receptor tyrosine kinases (TAM RTK), a group of indirect receptors for PS, provide survival signals to tumor cells [47]. Therefore, targeted blockade of the PS-PSR axis holds promise as a potential anti-tumor strategy.

##### CRT/LRP

Calreticulin (CRT) functions as an important ligand on apoptotic cells by stimulating its receptor low-density lipoprotein-related protein (LRP) on the phagocytes, stimulating Rac-1 and causing engulfment (efferocytosis) of apoptotic cells [30]. Lin et al. found that tumor Stannio-calcin-1 (STC1) interacts with CRT and minimizes CRT membrane exposure, thus preventing CRT-directed phagocytosis [48]. 

##### SLAMF7

Both macrophages and tumor cells express SLAMF7. All SLAM-related receptors are homotypic receptors (i.e., self-ligands), except for SLAMF4 [49]. Unlike SLAMF3 and SLAMF4, SLAMF7 is an “eat me” signal. Chen et al. demonstrated that SLAMF7 is important for the phagocytosis of hematopoietic tumor cells via Mac-1 integrin [2].

#### 2.1.4. Specific Antigen-Mediated ADCP Signals

ADCP is a crucial mechanism that contributes to the anti-tumor effect of mAbs. The Fc segment of the mAb attaches to FcR on macrophages, while the Fab segment binds to the antigenic epitope of tumor cells, mediating macrophage phagocytosis of tumor cells. However, the deleterious role of ADCP macrophages in immunosuppression must not be ignored. It was shown that macrophages after ADCP inhibit NK cell-mediated antibody-dependent cell-mediated cytotoxicity (ADCC) and T cell-mediated cytotoxicity in breast cancers and lymphomas [50].

### 2.2. Phagocytic Ability of Macrophages

Expression of phagocytic receptors, regulation of phagocytic signaling pathways, and activation of macrophages all influence the phagocytic ability of macrophages (Figure 2 and Table 2).

#### 2.2.1. Phagocytic Receptors

It is likely that professional phagocytes possess a greater phagocytic ability than nonprofessional phagocytes due to the presence of specific receptors that enhance the range of particles and phagocytic rate [16]. Macrophages recognize phagocyte-specific antigens and ligands through various receptors, such as complement receptors (CRs) and Fc receptors for IgG (FcγRs) [69,70]. When exposed to interferon-gamma (IFN-γ) and LPS, M1 macrophages express opsonic receptors, such as FcγRIII (CD16), but M2 macrophages express non-opsonic receptors more frequently (e.g., mannose receptors and scavenger receptors) [71]. 

Most particles are identified by multiple receptors, and these receptors can interact with each other and cause synergy. Various phagocytic receptors have dual functions that guide both adhesion and internalization, further complicating the link between these two related processes [16]. In addition, all these receptors induce rearrangements in the actin cytoskeleton that initiates internalization [16].

Furthermore, FcR-mediated phagocytosis can induce ADCP, and the activation of FcγRIIa (CD32A) and FcγRIIIa on macrophages is crucial for mediating ADCP [72]. 

Phagocytic receptors can guide macrophages to efficiently remove abnormal cells without accidentally injuring healthy cells. Many phagocytic signaling molecules are shared by both FcR and CR (e.g., tyrosine kinase, protein kinase C). Phagocytic signaling transmission eventually influences intracellular changes in macrophages.

#### 2.2.2. Activation of Macrophages

Macrophage activation and its increased phagocytic ability are closely related. Resting macrophages (M0) are commonly activated into two following phenotypes: (1) M1 or classically activated phenotype, which is activated by LPS or in combination with Th1 cytokines; (2) M2 or alternatively activated phenotype, which is activated by Th2 cytokines [73]. It is widely believed that M1 macrophages have stronger anti-tumor properties. Many experiments have achieved remarkable tumor-killing effects by polarizing macrophages to the M1 phenotype to promote phagocytosis. Moreover, a growing body of research demonstrates that macrophage phagocytic capacity is activated by some other substances (Figure 2 and Table 3). For example, the famous macrophage classical agonist LPS was proven to be the Toll-like receptor (TLR9) agonist, which is important for macrophage activation [74,75].

#### 2.2.3. Mitochondrial Fission

Mitochondrial fission plays a crucial role in harnessing macrophages to engulf tumors [85]. The increase in cytosolic calcium caused by mitochondrial fission prevents the phase transition of the Wiskott–Aldrich syndrome protein (WASP) into the Wiskott–Aldrich syndrome interacting protein (WIP) complex and allows PKC-θ to phosphorylate WIP [86]. Overexpression of GFPT2, an enzyme participating in the metabolism of glutamine, contributes to less available nutrients to stimulate mitochondrial fission, deters access of PKC-θ to compartmentalized WIP, and restrains the assembly of the phagocytic apparatus, thereby resisting the phagocytosis [85,86]. Li et al. demonstrate that mitochondrial dynamics regulate the phase transition of phagocytic apparatus and proposed GFPT2 to promote antibody-therapy [86].

## 3. Clinical Translation of Therapeutic Strategies Targeting Macrophage Phagocytosis Pathways

### 3.1. Therapeutic Applications Targeting Phagocytic Signals

**Figure 3:**
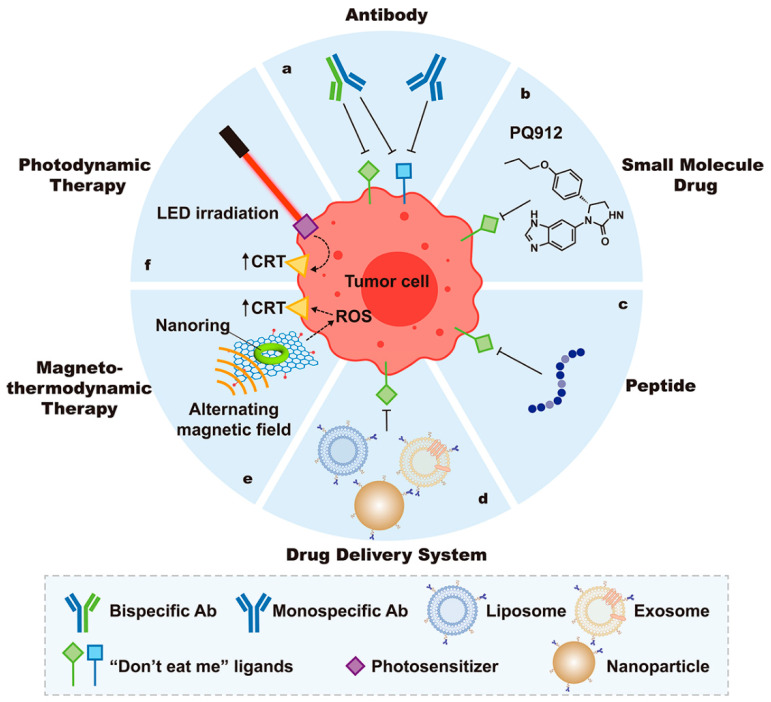
Strategies for strengthening macrophage-mediated phagocytosis based on phagocytosis signal regulation. To promote macrophage-mediated phagocytosis, “don’t eat me” signals are blocked using monospecific or bispecific antibodies (**a**), small molecule drugs (**b**), and peptides (**c**). Nanomaterials such as liposomes, exosomes, and nanoparticles are used as drug delivery systems (**d**) to carry therapeutics that encourage macrophage phagocytosis by blocking “don’t eat me” signals. (**e**) In magneto thermodynamic therapy, an increased level of ROS induces expression of the “eat me” signal CRT on tumor cells, which enhances macrophage-mediated phagocytosis. (**f**) Under LED irradiation, photosensitizers increase CRT on the surface of tumor cells, resulting in macrophage-mediated phagocytosis of tumor cells.

As previously mentioned, “find me”, “eat me”, and “don’t eat me” signals regulate macrophage-mediated phagocytosis [87]. Therapeutic strategies have been developed to promote phagocytosis activity by targeting phagocytic signals (Figure 3). The clinical trials of anti-tumor therapy harnessing macrophage mediated-phagocytosis are summarized in Table S1.

#### 3.1.1. Strategies Targeting “Find Me” Signals

“Find me” signals attract macrophages to migrate and initiate phagocytosis. For instance, it has been observed that GATA6 large peritoneal macrophages (GLPMs) invade growing metastatic tumors through the “find me” signal ATP, facilitating their progression [88]. Therefore, based on the “find me” signals’ properties of attracting phagocytes, we speculate that utilizing “find me” signals may be a potential anti-tumor strategy by (1) attracting TAMs away from the TME to reduce its pro-tumor effect; (2) combining with other strategies (e.g., mAbs and magnetic navigation) to go deep into tumor cells and slowly release the “find me” signals to recruit normal macrophages to engulf tumors. 

#### 3.1.2. “Don’t Eat Me” Signal Blockade

##### Monospecific Antibodies

The applications of “don’t eat me” signal-blocking mAb in anti-tumor therapy have come to light in recent studies, eliciting anti-tumor responses and causing positive changes in TME (Figure 3a). The “don’t eat me” signal CD47-SIRPα axis is a well-liked target in the development of anti-tumor therapeutics [89]. Anti-CD47 can selectively target tumor cells since tumor cells express CD47 at high levels [90]. Formerly known as hu5F9-G4, magrolimab has been demonstrated to bind to CD47 and facilitate efficient macrophage-mediated phagocytosis of tumor cells [91,92,93,94]. Besides hu5F9-G4, other anti-CD47s, including CC-90002 [95], AMMS4-G4 [96], AO-176 [96], IBI188 [97], SRF231 [98], 2C8 [99] were also developed. Although CD47 blockade has a good prospect as anti-tumor medicine, there are issues to be solved. The limited therapeutic impact of CD47-SIRPα monotherapy at the maximal tolerable dosage is clear according to early clinical studies [100,101]. In addition, the broad expression of CD47 in the human body causes an “antigen sink” effect that might reduce the therapeutic efficacy of CD47 blockades and patients need repeated injections of high-dose CD47 to obtain adequate drug exposure [102,103]. Moreover, CD47 blockade is associated with substantial adverse effects, most notably anemia, since CD47 is widely expressed in healthy cells, especially in red blood cells (RBCs) [100,104]. Furthermore, CD47 blockade or knockout is not sufficient to trigger phagocytosis sometimes, which requires opsonizing antibodies and surface exposure of “eat me” signals to serve as additional pro-phagocytic stimuli [94]. It is anticipated that using anti-SIRPα to target the SIRPα/CD47 axis may have a beneficial safety profile due to SIRPα’s more constrained expression, which can lower the risk of adverse events such as acute anemia, thrombocytopenia caused by anti-CD47 [105]. At present, over 10 CD47/SIRPα blockade drugs have advanced to active phase I/II/III clinical trials, indicating great clinical potential with published results [91,92,93,95,106,107,108].

Apart from the CD47-SIRPα axis, there are studies targeting other “don’t eat me” signals with different characteristics. Anti-CD47 G7mAb was developed by He et al., which selectively targeted HCC in vitro and in vivo [109]. To improve the internal stability and targeting accuracy of anti-CD24, they created and synthesized the NO donor HL-2, and conjugated HL-2 with anti-CD24 through a thioether bond, which they termed HN-01 [110]. The possible benefit of targeting CD24 stems from its absence on RBCs. Hence, it would not cause anemia [105]. Its expression on immune cells, including B-cells, neutrophils, neurons, and epithelial cells, however, might have negative side effects [105]. The aforementioned points raise the issue of antigen sinks and safety concerns.

Formerly known as a T cell immune checkpoint, the PD-1/PD-L1 axis has been targeted in clinical trials [111,112,113,114], which is now found to regulate macrophages. The expression of PD-L1 on tumor cells may facilitate avoidance of T cell cytotoxicity and macrophage-mediated phagocytosis, showing that the interruption of this pathway could unleash anti-tumor immunity through both adaptive and innate responses [115]. However, less than 30% of patients respond to anti-PD-1/PD-L1 therapy due to primary resistance [116]. In addition, the toxicity issue merits consideration, which may harm organs as a result of the induction of immune cells to target healthy tissues [117,118,119,120,121].

The “don’t eat me” signal blockades based on mAbs are promising anti-tumor therapies. Compared with other strategies, they have more clinical trials and results, showing preferable safety and practicality. To compensate for the deficiency of mAb to block “don’t eat me” signals, several strategies have emerged.

##### Bispecific Antibodies

Bispecific antibodies (BsAbs) can recognize and attach to two distinct antigens or epitopes to increase the effectiveness of treatment and lower the risk of unfavorable outcomes [122,123] (Figure 3a). CD47-targeted BsAbs could be a potential tactic to overcome CD47 blockade limits and further improve security and effectiveness [123,124].

The first anti-CD47/PD-L1 BsAb IBI322 uses a “1 + 2” configuration and a knobs-into-holes technique. IBI322 had a reduced binding affinity to CD47 versus a greater binding to PD-L1. This “imbalanced” design was anticipated to block CD47 on CD47^+^/PD-L1^+^ tumor cells more specifically than standard anti-CD47 while reducing on-target damage in normal tissues [103]. IBI322 is being studied in a phase I dosage escalation trial (NCT04328831). However, there have been no clinical data disclosed for it so far. 

NI-1701(NCT04806035) was designed by Buatois et al. It combines a high-affinity CD19-targeting arm with a variety of CD47-blocking arms. To balance the effectiveness of CD19^+^ cells against “off-target” effects, the CD47 arms were chosen with an affinity [125]. While BsAbs show therapeutic benefits in vitro and have reduced activity toward RBCs, these tumor antigen-directed methods face difficulties since the majority of their targets are only overexpressed rather than truly tumor-specific. Hence, BsAbs will also target cells that avidly express healthy target antigens, such as healthy cardiomyocytes when targeting Her2 or healthy epithelial cells when targeting EGFR [100].

##### Small-Molecule Drugs

To better the therapeutic benefit of inhibiting the CD47-SIRPα axis, small compounds offer special advantages (Figure 3b). Small-molecule drugs can not only boost the distribution of drugs in tissue and solid tumors and enhance bioavailability to make patients more convenient but they can also improve the half-life and lessen adverse reactions, enabling better management of effectiveness and toxicity [126].

Golgi-resident enzyme isoQC, which is absent in mature erythrocytes, is the essential regulator of pGlu modification of CD47 N-terminal peptide, which influences the interaction between CD47 and SIRPα [127]. To overcome anti-CD47-induced anemia, Wu et al. investigated the potential of using isoQC inhibition. Recent research has shown how effective the regulation of the CD47 pyroglutamate production by glutaminyl cyclase isoenzyme (QPCTL) inhibitors is in preventing CD47-SIRPα interaction [128]. By screening small molecules acquired from a library of naturally occurring compounds, they found Luteolin, a novel lead chemical of isoQC inhibitor [129]. Their findings demonstrated that treatment with luteolin would not result in erythrocyte destruction, which mended the deficiency of CD47 blockade. However, Luteolin may have poor oral bioavailability compared to that of PQ529 (a known isoQC inhibitor) [130], which may cause limited clinical translations of isoQC inhibitors currently. PQ912 is another small-molecule drug. Only PQ912 made it into the clinical trials (NCT02190708, NCT04498650) out of all the QPCTL inhibitors that were studied. In order to bypass the time-consuming cell engineering procedure, Li et al. devised an injectable gel containing therapeutic medicines that may be injected into the surgically resected wound site. The simple manufacture and administration, cheap cost, superior performance, and minimum toxicity make this gel highly practicable [131].

##### Peptides

Peptides have a variety of advantageous characteristics, including great target selectivity, minimal toxicity, and exceptional effectiveness, serving as a potential strategy for “don’t eat me” signal blockades [132] (Figure 3c). Through high-throughput phage display library bio-panning, Wang et al. discovered the new peptide pep-20, which selectively targets CD47 and blocks CD47/SIRPα axis [133]. RS17, a different CD47-targeted peptide, was created by using MOE analysis and is demonstrated to selectively bind to CD47’s extracellular domain [134]. Due to their smaller size compared to larger biomolecules, peptides have higher tissue penetration and lower systemic toxicity concerns [118,119]. Additionally, since peptides are simple to generate and store, they could be artificially changed at a minimal cost. Peptide medicines act as vaccines or therapeutic carriers, inducing tumor cell death and preventing angiogenesis, which have demonstrated distinct benefits and broad application potential [133,134].

##### Nanomedicine

Interest in nanomedicine applied to anti-tumor treatment has grown rapidly [135]. A variety of therapeutic medications, spanning small molecules to macromolecular compounds, have been effectively delivered utilizing nanomaterial-based delivery systems in preclinical and clinical trials for anti-tumor treatment [135] (Figure 3d). The therapeutic impact of anti-CD47 in vivo may be considerably increased and several biological obstacles might be surmounted by applying nanomaterials as delivery systems for “don’t eat me” signal blockades [136].

Given their small size, strong affinity, and good stability, nanobodies (Nbs) have been acknowledged as more suitable building blocks for the creation of innovative medicines in comparison to traditional mAbs [137,138]. By conducting four rounds of phage display bio-panning, Ma et al. screened the CD47-specific Nbs and created a new Nb fusion protein HuNb1-IgG4. HuNb1-IgG4 not only significantly enhanced the clearance of tumor cells, but also resulted in no agglutination of RBCs in vitro and exhibited high safety for the hematopoietic system in cynomolgus monkey [139].

It is generally known that nanoparticle-based drug delivery is a desirable approach for anti-tumor therapy [140]. To maximize drug release at the tumor locations, an ideal nanocarrier must possess special qualities such as strong biocompatibility under physiological settings and lengthy blood circulation by eluding the mononuclear phagocyte system’s detection [141].

Lipid-based nanoparticles are one of the most commonly employed delivery methods in nanomedicine due to their biocompatibility and capacity to convey a variety of substances, including proteins and therapeutic genes [142]. Ramesh et al. described a multivalent phagocytosis nano enhancer that may concurrently engage macrophages and tumor cells as a multivalent lipid-based platform consisting of phosphatidylcholine, DSPE-PEG-carboxylic acid, and cholesterol [143].

Exosomes are membrane-bound, nanoscale extracellular vesicles that can be released by a variety of cell types, and contain an enriched content of tiny molecules, proteins, and nucleic acids [144]. They have a wide range of exceptional qualities, including good biocompatibility, almost low immunogenicity, extended circulation, and non-toxicity. The benzoic-imine bonds of the nano-bioconjugates are destroyed in the acidic TME to produce anti-SIRPα and anti-CD47, which can inhibit SIRPα on macrophages and increase macrophage phagocytosis. In the meantime, native M1 exosomes successfully re-educate pro-tumoral M2 macrophages to anti-tumoral M1 macrophages [145]. Poor cargo encapsulation in exosomes, however, could limit their potential for drug delivery [146]. Given these difficulties, hybrid membrane nanovesicles or biomimetic nanovesicles have generated a great deal of attention in recent developments [147]. To create the hybrid nanovesicles, exosomes or cell membrane vesicles were combined with liposomes, which endows liposomes with biogenic capabilities [148]. Cheng et al. produced CD47-overexpressed hybrid nanovesicles by fusing gene-engineered exosomes with drug-loaded thermosensitive liposomes, which integrated photothermal therapy (PTT) with immunotherapy for the anti-tumor treatment by blocking CD47 [141].

In addition to the previously mentioned nanoparticle-based delivery methods, other nanotechnology-based immunotherapies that target macrophage phagocytosis have been developed, such as therapies using protein nanoparticles [149], polymeric nanoparticles [150,151], and inorganic nanoparticles [152,153].

#### 3.1.3. Exposure of “Eat Me” Signals

As previously mentioned, “eat me” signals are up-regulated on the tumor surface to harness the engulfment of tumor cells, shedding light on the likelihood of “eat me” signals serving as a target for the treatment.

One approach is to regulate the exposure of “eat me” signals on tumor cells, inducing phagocytosis and anti-tumor immune responses. According to Liu et al., CRT expression on 4T1 breast cancer cells was induced by ferrimagnetic vortex-domain iron oxide nanoring (FVIO)-mediated mild magnetic hyperthermia, which also encouraged the immune system’s phagocytic absorption of tumor cells [154] (Figure 3e). To solve the restricted therapeutic effectiveness of the magnetothermal (MTT) therapy, Liu et al. merged the MTT effect and the immunologic effect linked to reactive oxygen species (ROS). It was accomplished by creating a complex FVIO and graphene oxide (FVIOs-GO) hybrid nanoparticle as an effective magneto thermodynamic agent [155]. 

Mannose-conjugated-chlorin e6 (M-chlorin e6), a novel photosensitizer developed by Kimura et al., targets mannose receptors that are highly expressed on tumor cells and M2-TAMs (Figure 3f). In prior studies, they showed how M-chlorin e6 photodynamic therapy (PDT) decreased tumor volume and the percentage of M2-TAMs. The current study indicates that M-chlorin e6 PDT augments CRT expression on the tumor cell surface, causing macrophage-mediated phagocytosis [156].

Apart from the two methods above, “eat me” signals can be used to target TAM for precise engulfment of tumor cells, which was inspired by the mechanism of PS-mediated phagocytosis of apoptotic cells. The matrix metalloproteinase 2 (MMP2)-sensitive PS-modified nanoparticles (PSNP) were created to implement this concept. The NPs’ ability to phagocytose depends critically on the PS concentration, and spectacular phagocytosis was only seen when the PS content exceeded 75%. The NP formulation of the MMP2-sensitive PSNPs was enhanced to guarantee minimal phagocytosis in the absence of MMP2 and maximal phagocytosis in the presence of MMP2. The nanoparticles will not externalize PS to their surface until they get close to the MMP2-overexpressing tumor, which enables TAM-specific phagocytosis. This TAM selectivity was effectively replicated in zebrafish and tumor-bearing mice, among other biological models [157]. 

### 3.2. ADCP-Potentiating Agents

**Figure 4:**
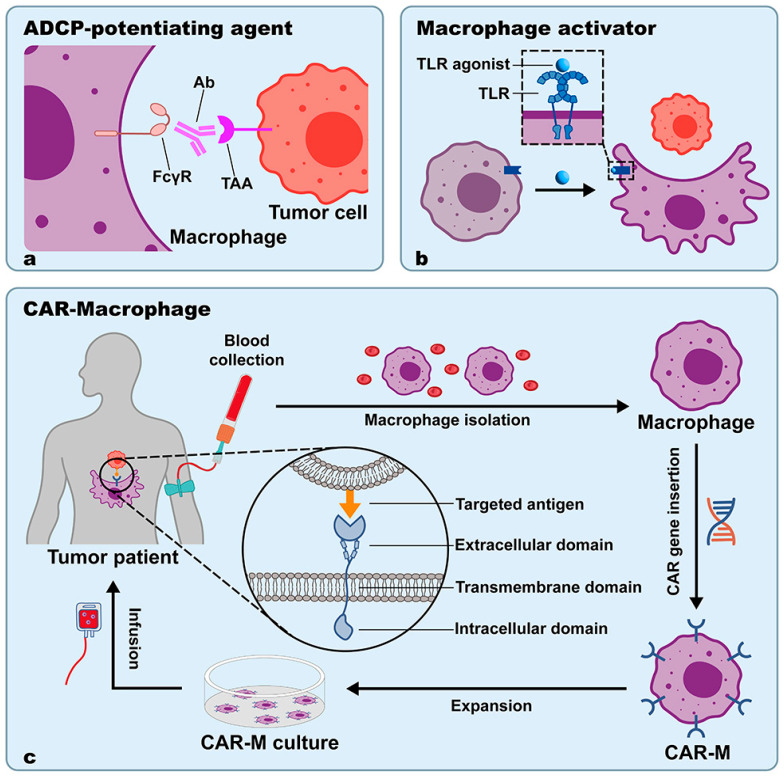
Strategies for strengthening macrophage-mediated phagocytosis based on phagocytosis ability regulation. (**a**) Using tumor-specific mAbs as ACDP-potentiating agents to induce macrophage-mediated ADCP. (**b**) Using macrophage activators to switch macrophages into a phenotype with greater capacity to phagocytose tumor cells. (**c**) Macrophages are collected from tumor patients’ blood and are designed to express CARs. After cell expansion, CAR-Ms are given back to patients through infusion. CARs detect and bind to targeted antigens on tumor cells, resulting in enhanced macrophage phagocytosis.

Tumor-specific mAbs can help to bypass the anti-phagocytic signals by targeting tumor cells and interacting with macrophage FcγR to cause ADCP [158] (Figure 4a). The potential of four mFcγRs to support macrophage phagocytosis of opsonized tumor cells and tumor growth suppression in vivo was proven by Chen et al. The results showed that while activating receptors (mFcγRI, mFcγRIII, and mFcγRIV) were equally capable of triggering specific tumor cell phagocytosis, the inhibitory receptor mFcγRII was unable to do so [159]. Some “don’t eat me” signal blockades, such as RF231(a fully human anti-CD47), can not only promote CD47-mediated death signaling in tumor cells but also triggers FcγR-mediated phagocytosis of tumor cells [98].

#### 3.2.1. Application Status

Clinically effective evidence suggests that many therapeutic antibodies’ anti-tumor benefits are primarily mediated by the stimulation of macrophage-induced ADCP [160]. One of the first mAb treatments authorized for the treatment of multiple myeloma that has relapsed or become resistant to treatment is elotuzumab, a new IgG1 mAb [161,162]. The capacity of elotuzumab to stimulate macrophage-mediated anti-myeloma phagocytic activity by activating the FcγR is shown by Kurdi et al. According to reports, elotuzumab also boosts macrophage activation in addition to enhancing macrophage concentration at the tumor location [161].

Trastuzumab and daratumumab are humanized mAbs and are in clinical development for anti-tumor treatment. One of the main modes of their action is antibody-dependent cell-mediated cytotoxicity (ADCC) mediated by NK cells. Both of them are now proven to induce ADCP and tumor cell death [163,164].

It has long been recognized that cyclophosphamide has significant immunomodulatory effects and that modest dosages can specifically kill regulatory T cells, which activate the immune system [155]. According to Naicker et al., cyclophosphamide alters the TME to stimulate macrophage recruitment, M1 polarization, and ADCP regulation, which independently boosts daratumumab-mediated tumor cell death [165].

#### 3.2.2. Advantages

The special qualities of anti-tumor mAbs are their target specificity, effectiveness, and low toxicity, which make these “magic bullets” essential in the armory used to combat tumors [160,166]. Improving ADCP is essential from the standpoint of immunotherapy because it can raise cross-presentation and subsequently prompt tumor-specific anti-tumor responses [167]. The robust capacity of mAbs to mediate ADCP of single target cells might be used to treat patients with solid tumors who have limited residual diseases. For instance, preoperative mAbs treatment may be extremely beneficial for patients having surgery to remove colorectal cancer by stopping the adhesion and proliferation of circulating tumor cells in the liver, which is correlated with bad prognoses for patients. Surgery can remove the main part of the tumor, while adjuvant mAbs treatment may cause ADCP of any tumor cells that are still present [168].

#### 3.2.3. Limitations and Outlooks

mAbs have limitations owing to their large size. In the context of treating solid tumors, they have shown poor penetration of the tumor tissue, with only 0.001–0.01% of the administered dose accumulating per gram of solid tumor [169]. According to a recent study, treatment with ADCP can result in the development of immunosuppressive macrophages with high expressions of PD-L1 and indoleamine 2,3-dioxygenase. This in turn induces compensatory immunosuppression that negatively affects both ADCC mediated by NK cells and immune response mediated by T cells. As a result, the anti-tumor effects brought about by macrophage-mediated phagocytosis during antibody treatment can be suppressed or even overwhelmed [50].

The immunosuppressive effects induced by ADCP imply that the combination with immune checkpoint blockades may be a viable strategy for maximizing antitumor immunity when using therapeutic mAbs [167]. Alternatively, it has been shown that blocking the CD47-SIRPα signaling pathway can collaborate with tumor-specific antibodies to enhance the clearance of tumor cells. Moreover, as to advanced-stage HER2^+^ breast cancer patients who develop resistance to trastuzumab and relapse, the resistance can be overcome by the combination with CD47 blockade in most cases [167].

Additional combination therapy regimens should also be considered. Xu et al. offered an explanation of how paclitaxel enhanced ADCP efficiency, thereby providing a promising method that utilizes conventional anti-tumor medications to promote macrophage phagocytosis and improve the effectiveness of therapeutic anti-tumor antibodies [158].

### 3.3. Macrophage Activators

TAMs have the ability to change their phenotypes in response to their precise position inside the tumor and the characteristics of their immediate microenvironment. In the majority of tumors, TAMs with tumor-promoting properties dominate the TME, resulting in poorer prognoses for patients [170]. The polarization of TAMs toward the M1 phenotype has been considered to improve phagocytosis, hence increasing the effectiveness of anti-tumor treatment in patients [171,172]. The reprogramming of macrophages to induce M1-like phenotypes while suppressing M2/TAM characteristics is a promising strategy for developing immunotherapeutic interventions against tumors [173] (Figure 4b).

#### 3.3.1. TLR Agonists

The application of TLR agonists has been shown to effectively impede tumor growth and significantly reverse the immunosuppression induced by ADCP, demonstrating their potential for re-educating TAMs [174]. Notably, TLR agonists may serve as a favorable immune adjuvant for anti-tumor treatment, as they enhance both the phagocytic and oxidative burst mechanisms of macrophages’ anti-tumor response with relatively low toxicity [82]. 

As reported by Zhang et al., G. atrum polysaccharide (PSG-1) demonstrated the ability to activate macrophages through TLR4-dependent signaling pathways. A small molecule TLR agonist, referred to as pyrimido [5,4-b] indole (PBI1) was reported to elicit anti-tumor immune responses and boost macrophage phagocytic efficacy by five times compared to non-treated macrophages [82]. Resiquimod (R848), a TLR7/8 agonist, was proven to induce polarization of the M2 phenotype toward the M1 phenotype [175]. A novel combination cancer immunotherapy was developed, involving the encapsulation of R848 into liposomes along with therapeutic antibodies. This approach facilitated the targeted delivery of R848 to TAMs, resulting in efficient re-education and enhanced response to ADCP [176]. Li et al. observed that the administration of oxaliplatin (OXA) and R848 together resulted in a synergistic anti-tumor effect, surpassing that of either agent used alone. This provided proof of the therapeutic potential of macrophage re-education in the chemotherapy of lung cancer [175].

A substantial part of clinical trials studying TLR agonists for use in anti-tumor therapy has paid attention to TLR9 [177,178], TLR7/8 [179], and TLR3 [180]. These clinical trials showed the great potential of TLR agonists in clinical application.

There are still certain restrictions of the immunotherapy using TAM re-education method. Firstly, sufficient contact areas between macrophages and tumor cells are important for ADCP, but TAMs are distributed diversely in tumors. TAMs are evenly dispersed in small tumors, but they are mostly located toward the edges of large tumors [181]. Secondly, the substantial variability in tumor growth inhibition and ADCP tests in vivo suggested that each patient responded differently to this medication. This may be due to the fact that the quantity and polarization states of TAMs as well as ADCP responses vary between different models. To determine whether a patient will benefit from this combination therapy, it is necessary to diagnose the quantity and polarization state of TAMs in precision medicine [176].

#### 3.3.2. CSF-1 Inhibitors

With CSF-1R suppression, glioma TAMs lose their M2 characteristics and exhibit increased phagocytosis behaviors, as shown by Pyonteck et al. [83]. A CSF-1R inhibitor (BLZ945) that may cut off the CSF1-CSF1R pathway and decrease M2 phenotypes was recently produced by Fang et al. using a magnetic liposomal system modified with cell-penetrating TAT peptide (termed TAT-BLZmlips) [182]. TAT-BLZmlips have been shown to pierce the tumor’s interior and have higher tumor permeability. Both histopathological analysis and bodyweight monitoring revealed no overt side effects. It should be highlighted that the group of TAT-BLZmlips had higher drug distribution to the liver, and more research is required to explain this phenomenon [182].

#### 3.3.3. HDAC Inhibitors

The histone deacetylase (HDAC) inhibitor termed TMP195 stimulated myeloid cells to have strong phagocytic activity [84]. Moreover, TMP195 can inhibit colorectal cancer growth by polarizing M1 macrophages [183]. Yue et al. have created polydopamine NPs that were employed as TMP195 delivery agents and photothermal transduction agents to concurrently cauterize tumor cells and regress the residual tumors following PTT. 

These biomimetic nanoparticles greatly raised the number of M1-like TAMs in the breast tumor model, leading to a tumor-elimination rate of 60%, up from 10% following PTT. It is important to note that after decorating the macrophage membrane with nanoparticles, the drug loading effectiveness was marginally reduced [184].

### 3.4. CAR-M

Chimeric Antigen Receptor T-Cell (CAR-T) treatment has been proven to have significant pre-clinical success in treating hematological malignancies. However, it has limited effectiveness in treating solid tumors [185,186]. Limited CAR-T cell penetration into solid tumors and CAR-T cell inactivation by the TME are the two factors resulting in restricted therapeutic efficacy. Moreover, CAR-T treatment frequently comes with fatal toxicities, such as cytokine release syndrome [187]. Employing macrophages modified with CAR (CAR-M) to treat solid tumors is expected since they can interact with practically all cellular components in the TME and infiltrate solid tumor tissue [188]. Structurally, the CAR consists of three functional components: an antigen-recognition domain, usually a single-chain variable fragment (scFv) derived from a mAb that targets the selected antigen (i.e., CD19 and HER2); a hinge domain (typically CD8) that connects the recognition site to the transmembrane domain which bridges the membrane; and an intracellular domain that presents dedicated downstream signaling [189] (Figure 4c). 

#### 3.4.1. Application Status

Employing CAR for macrophages is still in its early stages. Morrissey et al. designed a set of CARs for Phagocytosis (CAR-Ps) aimed at augmenting phagocytic processes. The intracellular domains of both Megf10 and FcγR have been found to robustly trigger engulfment in a manner independent of their respective native extracellular domains [190]. Klichinsky et al. reported a CAR that produced a persistent M1 subtype, which effectively overcame the intrinsic resistance of primary human macrophages against genetic engineering, showing efficacy in antigen-specific phagocytosis and clearance of tumor cells in vitro [191]. Zhang et al. created CAR-iMacs, which can selectively engulf tumor cells and have antigen-dependent functions [192]. They converted peripheral blood mononuclear cells (PBMCs) into induced pluripotent stem cells (iPSCs) by reprogramming them using non-integrating episomal vector encoded reprogramming factors. These iPSC-derived CAR-macrophages possess an M2 phenotype and convert to a pro-inflammatory M1 phenotype upon encounter with target cells. Subsequently, Zhang et al. engineered CAR into iPSCs via lentiviral transduction and established a protocol for myeloid/macrophage differentiation to induce CAR-iPSCs toward myeloid cell lineages, thereby enabling the unlimited production of engineered macrophage cells [87]. Kang et al. employed macrophage-targeting polymer nanocarriers to transport genes expressing CAR and IFN-γ genes to macrophages in vivo in order to produce CAR-M1 macrophages that are able to execute CAR-mediated tumor phagocytosis [187].

#### 3.4.2. Advantages

First, due to the physical obstacles created by the matrix enclosing the tumor cells, T-cells are incapable of penetrating the TME, while macrophages immerse in the TME significantly [186]. It was proven that macrophages can migrate into the TME when they detect hypoxia status and associated byproducts [185]. The application of CAR-M can improve anti-tumor therapy by lowering the percentage of TAMs and changing their phenotype [123,193]. Secondly, CAR-M can improve antigen presentation and thereby increasing the cytotoxicity of T cells. Furthermore, CAR-M has a shorter circulation duration and lower normal tissue toxicity pared with CAR-T. 

#### 3.4.3. Limitations and Outlooks

Although widely acknowledged, many of the drawbacks of CAR-M are still unknown due to the characteristics of macrophages and research status. First of all, the proliferation of macrophages has not been observed either in vitro or in vivo following injection. It should be noted that the therapeutic efficacy may be impacted as patients can only take a certain number of macrophages [194]. Secondly, following injection, exogenous macrophages traverse the lung and subsequently accumulate primarily in the liver, which may have negative implications for anti-tumor treatment efficacy [195]. Additionally, the persistence of CAR-M cells is also worthy of attention, and a series of apoptotic markers expressed on macrophages appear to warrant careful investigation [196]. Overcoming potential barriers to the effective trafficking and persistence of CAR-M cells within solid tumors presents a critical challenge for future therapeutic approaches [196].

During the clinical implementation process of CAR-M treatment, the complicated immunological microenvironment should be taken into account [123]. The majority of targeted tumor antigens are frequently expressed in certain populations of healthy cells, which may result in off-target harm [189]. Only three clinical trials (NCT03608618, NCT05007379, NCT04660929) have been launched until now, and no results were reported. More clinical trials are required to confirm the safety and efficiency of CAR-M and explore the limits that have yet to be discovered. Furthermore, the combination of CAR-M therapy with other forms of immunotherapy serves as a feasible approach. The “don’t eat me” signal blockades may augment the phagocytosis of CAR-M. The combined use of CAR-M with CAR-T presents a potential therapeutic option for patients with solid tumors of high burden [188].

## 4. Perspectives and Conclusions

**Figure 5:**
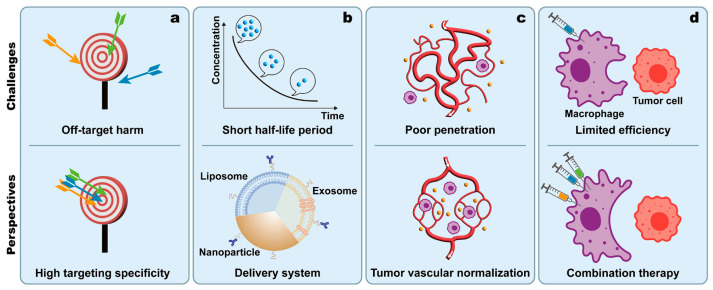
Current challenges and future perspectives for the application of macrophage phagocytosis-promoting therapy. (**a**) Off-target harm leads to adverse effects and raises safety concerns, which raises the demand for higher targeting specificity. (**b**) The short half-life period of drugs or CAR-Ms results in limited effects on promoting macrophage phagocytosis. Delivery systems are needed to prolong the circulation time of drugs and CAR-Ms. (**c**) Abnormal vessels cause poor penetration of drugs and CAR-Ms into solid tumors, which can be facilitated by vessel normalization. (**d**) The limited efficiency of harnessing macrophage-mediated phagocytosis can be improved by combination therapy.

In general, this review summarized the mechanisms and influencing factors of macrophage-mediated phagocytosis. It demonstrated the formidable roles of macrophages in anti-tumor therapy. All kinds of anti-tumor strategies based on harnessing macrophage-mediated phagocytosis have promising prospects, yet they still face challenges in certain aspects (Figure 5). 

Firstly, instead of pointing to target cells (tumor cells or macrophages) specifically, anti-tumor drugs based on harnessing macrophage-mediated phagocytosis may accidentally attack healthy cells and cause off-target harm (Figure 5a). It then leads to adverse effects and raises safety concerns, such as the anemia brought about by anti-CD47. Accordingly, BsAbs and nanomedicines are now applied as multi-targeting strategies. They improve the targeting specificity of drugs and lower the risk of adverse events [125,152]. Future research should aim at more specific targets and more precise identification of tumor cells. Secondly, the short half-life period of drugs or CAR-Ms leads to limited effects on harnessing macrophage-mediated phagocytosis [196] (Figure 5b). This problem can be solved by equipping the drugs or CAR-Ms with “armors”, the drug delivery systems across the nano, micro, and macro scales, which are associated with an extended half-life [197]. Thirdly, the poor penetration of drugs is always an insurmountable difficulty in solid tumors (Figure 5c). As for CAR-M, its penetration into solid tumors is better than CAR-T, but still not in the ideal situation. Highly abnormal and dysfunctional vasculature of tumors leads to an elevated interstitial fluid pressure, which impedes the homogeneous distribution of therapeutic agents throughout the tumor volume [198]. Studies have shown that normalization of the tumor vasculature can overcome the physical barrier to drug transport and improve immune effector cell infiltration [198,199]. The mechanism of vessel normalization is to decrease tumor interstitial fluid pressure as well as increase perfusion and oxygenation, which can be achieved by utilizing therapeutic blockades of proangiogenic factors [200,201]. Moreover, the efficiency of promoting macrophage-mediated phagocytosis is limited and needs improvement (Figure 5d). To this end, a combination of strategies that promote macrophage-mediated phagocytosis in multiple pathways may achieve a synergistic effect.

Overall, the current research direction mainly focuses on the advancement of phagocytosis, the reduction of adverse effects and the improvement of patient outcomes. In addition to optimizing existing strategies, it is important to find more specific macrophage-related molecules. Taken together, anti-tumor strategies based on harnessing macrophage-mediated phagocytosis may provide novel therapeutic options for future cancer treatment.

## Figures and Tables

**Table 1:** Phagocytic signals regulating macrophage-mediated phagocytosis.

Phagocytic Signals	Ligands	Targets	Effects on Phagocytosis	Refs
Nucleotides (ATP, UTP)	P2Y_2_	Apoptotic cells	Promote P2Y_2_-dependent recruitment of phagocytes	[20]
CX3CL1	CX3CR1	Bacteria	Control the clearance of entero-invasive pathogens by DCs	[21]
LPC	G2A	Apoptotic cells	Migrate macrophages toward LPC	[22]
S1P	GPCR	Apoptotic cells	Attract phagocytic cells	[23,24]
RP S19	C5aR	Apoptotic cells	Migrate monocytes/macrophages	[25]
PS (PtdSer)	PSR (e.g., stabilin-2)	Apoptotic cells	Stimulate membrane ruffling, vesicle formation, “bystander” uptake of cells, promote clearance	[26,27]
CRT	LRP	Viable or apoptotic cells	Initiate clearance	[28,29,30]
CD47	SIRPα	Opsonized RBCs, etc.	Regulate complement-mediated phagocytosis	[31]
SLAMF3 and SLAMF2	Specific SFR members, mainly SLAMF3 and SLAMF4	Hematopoietic cells	Inhibit “eat me” signals, mitigate macrophage phagocytosis, regulate signals transduced by TLR4	[32,33]
CD24	Siglec-10	Tumor cells	Block cytoskeletal rearrangement	[34]
PD-1	PD-L1	Tumor cells	Inhibit phagocytosis	[35,36]
MHC-I	LILRB	Cancer cells	Inhibit phagocytosis	[37]

Abbreviations: LPC, Lysophosphatidylcholine; DC, Dendritic cells; S1P, Sphingosine-1-Phosphate; GPCR, G-Protein-Coupled Receptor; RP S19, Ribosomal Protein S19; C5aR, C5a Receptor; PS, Phosphatidylserine; CRT, Calreticulin; LRP, Lipoprotein-Related Protein; SIRPα, Signal-Regulatory Protein α; RBC, Red blood cell; SLAMF, Signaling Lymphocytic Activation Molecule Family; SFR, SLAM family receptors; TLR4, Toll-Like Receptor 4; Siglec-10, Sialic-acid-binding Ig-like lectin 10; PD-L1, Programmed Death-Ligand 1; LILRB1, Leukocyte immunoglobulin-like receptor B1.

**Table 2:** Phagocytic receptors and pathways.

Receptors	Ligands	Downstream Signaling Molecules	Mechanisms	Refs
CD44	/	Src family kinases, Syk, Rac1, PI-3K, Rho GTPases	Internalize large particle, induce mature phagosome formation	[51]
FcγR	IgG-opsonized particles	CAPRI, Cdc42, Rac, Rho	Internalize, recruit actin and Arp2/3 complex	[52,53,54,55]
		Cdc42, WASp	Recruit P-Tyr proteins into the phagocytic cup, possibly assemble a regulated cytoskeletal complex at specialized sites of actin polymerization	[56]
		PKC-ε	Regulate vesicle delivery and focal exocytosis	[57]
		Lyn and Hck (Src family kinases), Syk kinase, PI3K, PI(3,4,5)P3	Assemble a complex of proteins around the FcR	[58,59,60]
FcR	IgG-opsonized particles	Bcl10, vesicular OCRL phosphatase	Complete the phagosome closure, regulate PI(4,5)P2 and F-actin turnover	[61,62]
		TI-VAMP /VAMP7	Control exocytosis and membrane extension	[63]
		ARF6	Regulate membrane recycling	[64]
FcγR; CR	IgG-opsonized particles, complement-opsonized particles	PLC and PLD, Ca^2+^, InsP3 and S1P-SOCE channels, cytosolic Ca^2+^ elevation	Promote the actin meshwork solubilization, and phagosomes fusion with granules containing lytic enzymes, the assembly and activation of the superoxide-generating NADPH oxidase complex	[65]
CR3	C3bi/complement-opsonized particles	Arp2/3 complex, Rho	Regulate actin assembly	[52,55]
SR	Effete components, such as apoptotic cells	Microtubules, PKC, tyrosine, MAPK, PI3K	/	[66,67]
Dectin-1	Fungal β-glucan	BTK, Vav1, PLCγ2	Ensue F-actin formation, participate in DAG production	[68]

Abbreviations: Syk, Spleen tyrosine kinase; PI3K, Phosphatidylinositol 3-Kinase; Rho, Ras-Homolog; FcγR, Fcγ Receptor; CAPRI, Calcium-Promoted Ras Inactivator; Cdc42, Rho family GTPase; Arp2/3 complex, Actin-Related Protein 2/3 complex; WASp, Wiskott–Aldrich protein; P-Tyr proteins, Tyrosine Phosphorylated Proteins; PKC-ε, Protein Kinase C-ε; PI(3,4,5)P3, PI-3,4,5-trisphosphate; Bcl10, B cell lymphoma/leukemia-10; OCRL, Oculocerebrorenal syndrome of Lowe 1; F-actin, Fibrous actin; PI(4,5)P2, Phosphatidylinositol 4,5-bisphosphate; TI-VAMP, TeNT-Insensitive Vesicle-Associated, soluble *N*-ethylmaleimide-sensitive factor attachment protein receptors protein; ARF6, ADP Ribosylation Factor 6; CR, Complement Receptors; PLC, Phospholipase C; PLD, Phospholipase D; InsP3, Inositol trisphosphate; S1P, Sphingosine-1-Phosphate; SOCE, Store-Operated Calcium Entry; SR, Scavenger Receptors; PKC, Protein Kinase C; MAPK, Mitogen-Activated Protein Kinases; BTK, Bruton’s Tyrosine Kinase; Vav1, guanine nucleotide exchange factor; PLCγ2, Phospholipase C gamma 2; PIs, Phosphoinositides; DAG, Diacylglycerol; PKC, Protein Kinase C.

**Table 3:** Phagocytic receptors and pathways.

Activators	Receptors	Targets	Critical Molecules or Pathways	Mechanisms	Refs
CD300b	PS	Apoptotic cells	Adaptor: DAP12	Accumulate in phagocytic cups, facilitate engulfment	[76]
LPS	TLRs	Bacteria or their components	Actin-Cdc42/Rac (Rho family GTPase) pathway, MyD88-p38 signaling pathway	Regulate phagocytosis, help phagocytes sense bacteria	[77,78]
CD40 agonist	CD40	Tumor cells	ERK1/2 pathway	Drive macrophage become tumoricidal, facilitate the depletion of tumor stroma	[79,80]
CpG	TLR9	Tumor cells	FAO	Increase ECAR, basal OCR, and total mitochondria, change the central carbon metabolism, engulf CD47^+^ cancer cells	[81]
PBI1	TLR4	Tumor cells	/	Enhance macrophage phagocytic efficiency five-fold	[82]
GSF-1R inhibitor	CSF-1R	Tumor cells	/	Enhance phagocytosis	[83]
HDAC inhibitor	HDAC	Tumor cells	/	Modulate macrophage phenotypes	[84]

Abbreviations: PS, Phosphatidylserine; ITAM, Immunoreceptor Tyrosine-based Activation Motif; DAP12, DNAX Activating Protein of 12 kDa; ECAR, Extracellular Acidification Rate; OCR, Oxygen Consumption Rate; HDAC, Histone deacetylase.

## Data Availability

Not applicable.

## Supplementary Materials

The following supporting information can be downloaded at: https://www.mdpi.com/article/10.3390/cancers15102717/s1, Table S1: Clinical trials of anti-tumor therapy harnessing macrophage mediated-phagocytosis. References [202,203,204,205,206,207,208,209,210,211,212,213,214,215,216,217,218,219,220,221,222,223,224,225,226,227,228,229] are cited in the Supplementary Materials.

## References

[1] Weiss G., Schaible U.E. (2015). Macrophage defense mechanisms against intracellular bacteria. Immunol. Rev..

[2] Chen J., Zhong M.C., Guo H., Davidson D., Mishel S., Lu Y., Rhee I., Perez-Quintero L.A., Zhang S., Cruz-Munoz M.E. (2017). SLAMF7 is critical for phagocytosis of haematopoietic tumour cells via Mac-1 integrin. Nature.

[3] Ma L., Li W., Zhang Y., Qi L., Zhao Q., Li N., Lu Y., Zhang L., Zhou F., Wu Y. (2022). FLT4/VEGFR3 activates AMPK to coordinate glycometabolic reprogramming with autophagy and inflammasome activation for bacterial elimination. Autophagy.

[4] Murray P.J., Wynn T.A. (2011). Protective and pathogenic functions of macrophage subsets. Nat. Rev. Immunol..

[5] Cavaillon J.M. (2011). The historical milestones in the understanding of leukocyte biology initiated by Elie Metchnikoff. J. Leukoc. Biol..

[6] Ma Y., Kemp S.S., Yang X., Wu M.H., Yuan S.Y. (2023). Cellular mechanisms underlying the impairment of macrophage efferocytosis. Immunol. Lett..

[7] Razi S., Yaghmoorian Khojini J., Kargarijam F., Panahi S., Tahershamsi Z., Tajbakhsh A., Gheibihayat S.M. (2023). Macrophage efferocytosis in health and disease. Cell Biochem. Funct..

[8] Weiskopf K., Weissman I.L. (2015). Macrophages are critical effectors of antibody therapies for cancer. MAbs.

[9] Ruffell B., Affara N.I., Coussens L.M. (2012). Differential macrophage programming in the tumor microenvironment. Trends Immunol..

[10] Nesbit M., Schaider H., Miller T.H., Herlyn M. (2001). Low-level monocyte chemoattractant protein-1 stimulation of monocytes leads to tumor formation in nontumorigenic melanoma cells. J. Immunol..

[11] Imbert P.R.C., Saric A., Pedram K., Bertozzi C.R., Grinstein S., Freeman S.A. (2021). An Acquired and Endogenous Glycocalyx Forms a Bidirectional “Don’t Eat” and “Don’t Eat Me” Barrier to Phagocytosis. Curr. Biol..

[12] Bingle L., Brown N.J., Lewis C.E. (2002). The role of tumour-associated macrophages in tumour progression: Implications for new anticancer therapies. J. Pathol..

[13] Cheng N., Bai X., Shu Y., Ahmad O., Shen P. (2021). Targeting tumor-associated macrophages as an antitumor strategy. Biochem. Pharmacol..

[14] Park J.B. (2003). Phagocytosis induces superoxide formation and apoptosis in macrophages. Exp. Mol. Med..

[15] Freeman S.A., Grinstein S. (2014). Phagocytosis: Receptors, signal integration, and the cytoskeleton. Immunol. Rev..

[16] Aderem A., Underhill D.M. (1999). Mechanisms of phagocytosis in macrophages. Annu. Rev. Immunol..

[17] Brown G.C., Neher J.J. (2012). Eaten alive! Cell death by primary phagocytosis: ‘phagoptosis’. Trends Biochem. Sci..

[18] Kelley S.M., Ravichandran K.S. (2021). Putting the brakes on phagocytosis: “don’t-eat-me” signaling in physiology and disease. EMBO Rep..

[19] Lauber K., Blumenthal S.G., Waibel M., Wesselborg S. (2004). Clearance of apoptotic cells: Getting rid of the corpses. Mol. Cell..

[20] Elliott M.R., Chekeni F.B., Trampont P.C., Lazarowski E.R., Kadl A., Walk S.F., Park D., Woodson R.I., Ostankovich M., Sharma P. (2009). Nucleotides released by apoptotic cells act as a find-me signal to promote phagocytic clearance. Nature.

[21] Niess J.H., Brand S., Gu X., Landsman L., Jung S., McCormick B.A., Vyas J.M., Boes M., Ploegh H.L., Fox J.G. (2005). CX3CR1-mediated dendritic cell access to the intestinal lumen and bacterial clearance. Science.

[22] Yang L.V., Radu C.G., Wang L., Riedinger M., Witte O.N. (2005). Gi-independent macrophage chemotaxis to lysophosphatidylcholine via the immunoregulatory GPCR G2A. Blood.

[23] Lee M.J., Van Brocklyn J.R., Thangada S., Liu C.H., Hand A.R., Menzeleev R., Spiegel S., Hla T. (1998). Sphingosine-1-phosphate as a ligand for the G protein-coupled receptor EDG-1. Science.

[24] Gude D.R., Alvarez S.E., Paugh S.W., Mitra P., Yu J., Griffiths R., Barbour S.E., Milstien S., Spiegel S. (2008). Apoptosis induces expression of sphingosine kinase 1 to release sphingosine-1-phosphate as a “come-and-get-me” signal. FASEB J..

[25] Nishiura H., Zhao R., Yamamoto T. (2011). The role of the ribosomal protein S19 C-terminus in altering the chemotaxis of leucocytes by causing functional differences in the C5a receptor response. J. Biochem..

[26] Hoffmann P.R., deCathelineau A.M., Ogden C.A., Leverrier Y., Bratton D.L., Daleke D.L., Ridley A.J., Fadok V.A., Henson P.M. (2001). Phosphatidylserine (PS) induces PS receptor-mediated macropinocytosis and promotes clearance of apoptotic cells. J. Cell Biol..

[27] Park S.Y., Jung M.Y., Kim H.J., Lee S.J., Kim S.Y., Lee B.H., Kwon T.H., Park R.W., Kim I.S. (2008). Rapid cell corpse clearance by stabilin-2, a membrane phosphatidylserine receptor. Cell Death Differ..

[28] Lillis A.P., Van Duyn L.B., Murphy-Ullrich J.E., Strickland D.K. (2008). LDL receptor-related protein 1: Unique tissue-specific functions revealed by selective gene knockout studies. Physiol. Rev..

[29] Kinchen J.M., Cabello J., Klingele D., Wong K., Feichtinger R., Schnabel H., Schnabel R., Hengartner M.O. (2005). Two pathways converge at CED-10 to mediate actin rearrangement and corpse removal in C. elegans. Nature.

[30] Gardai S.J., McPhillips K.A., Frasch S.C., Janssen W.J., Starefeldt A., Murphy-Ullrich J.E., Bratton D.L., Oldenborg P.A., Michalak M., Henson P.M. (2005). Cell-surface calreticulin initiates clearance of viable or apoptotic cells through trans-activation of LRP on the phagocyte. Cell.

[31] Oldenborg P.A., Gresham H.D., Lindberg F.P. (2001). CD47-signal regulatory protein alpha (SIRPalpha) regulates Fcγ and complement receptor-mediated phagocytosis. J. Exp. Med..

[32] Wang N., Satoskar A., Faubion W., Howie D., Okamoto S., Feske S., Gullo C., Clarke K., Sosa M.R., Sharpe A.H. (2004). The cell surface receptor SLAM controls T cell and macrophage functions. J. Exp. Med..

[33] Li D., Xiong W., Wang Y., Feng J., He Y., Du J., Wang J., Yang M., Zeng H., Yang Y.G. (2022). SLAMF3 and SLAMF4 are immune checkpoints that constrain macrophage phagocytosis of hematopoietic tumors. Sci. Immunol..

[34] Barkal A.A., Brewer R.E., Markovic M., Kowarsky M., Barkal S.A., Zaro B.W., Krishnan V., Hatakeyama J., Dorigo O., Barkal L.J. (2019). CD24 signalling through macrophage Siglec-10 is a target for cancer immunotherapy. Nature.

[35] Gordon S.R., Maute R.L., Dulken B.W., Hutter G., George B.M., McCracken M.N., Gupta R., Tsai J.M., Sinha R., Corey D. (2017). PD-1 expression by tumour-associated macrophages inhibits phagocytosis and tumour immunity. Nature.

[36] Patsoukis N., Duke-Cohan J.S., Chaudhri A., Aksoylar H.I., Wang Q., Council A., Berg A., Freeman G.J., Boussiotis V.A. (2020). Interaction of SHP-2 SH2 domains with PD-1 ITSM induces PD-1 dimerization and SHP-2 activation. Commun. Biol..

[37] Barkal A.A., Weiskopf K., Kao K.S., Gordon S.R., Rosental B., Yiu Y.Y., George B.M., Markovic M., Ring N.G., Tsai J.M. (2018). Engagement of MHC class I by the inhibitory receptor LILRB1 suppresses macrophages and is a target of cancer immunotherapy. Nat. Immunol..

[38] Ravichandran K.S. (2011). Beginnings of a good apoptotic meal: The find-me and eat-me signaling pathways. Immunity.

[39] Veillette A., Chen J. (2018). SIRPalpha-CD47 Immune Checkpoint Blockade in Anticancer Therapy. Trends Immunol..

[40] Zhang X., Wang Y., Fan J., Chen W., Luan J., Mei X., Wang S., Li Y., Ye L., Li S. (2019). Blocking CD47 efficiently potentiated therapeutic effects of anti-angiogenic therapy in non-small cell lung cancer. J. Immunother. Cancer.

[41] de Silva S., Fromm G., Shuptrine C.W., Johannes K., Patel A., Yoo K.J., Huang K., Schreiber T.H. (2020). CD40 Enhances Type I Interferon Responses Downstream of CD47 Blockade, Bridging Innate and Adaptive Immunity. Cancer Immunol. Res..

[42] Theruvath J., Menard M., Smith B.A.H., Linde M.H., Coles G.L., Dalton G.N., Wu W., Kiru L., Delaidelli A., Sotillo E. (2022). Anti-GD2 synergizes with CD47 blockade to mediate tumor eradication. Nat. Med..

[43] Abdel-Bar H.M., Walters A.A., Lim Y., Rouatbi N., Qin Y., Gheidari F., Han S., Osman R., Wang J.T., Al-Jamal K.T. (2021). An “eat me” combinatory nano-formulation for systemic immunotherapy of solid tumors. Theranostics.

[44] Lemke G. (2019). How macrophages deal with death. Nat. Rev. Immunol..

[45] Utsugi T., Schroit A.J., Connor J., Bucana C.D., Fidler I.J. (1991). Elevated expression of phosphatidylserine in the outer membrane leaflet of human tumor cells and recognition by activated human blood monocytes. Cancer Res..

[46] Sharma B., Kanwar S.S. (2018). Phosphatidylserine: A cancer cell targeting biomarker. Semin. Cancer Biol..

[47] Graham D.K., DeRyckere D., Davies K.D., Earp H.S. (2014). The TAM family: Phosphatidylserine sensing receptor tyrosine kinases gone awry in cancer. Nat. Rev. Cancer.

[48] Lin H., Kryczek I., Li S., Green M.D., Ali A., Hamasha R., Wei S., Vatan L., Szeliga W., Grove S. (2021). Stanniocalcin 1 is a phagocytosis checkpoint driving tumor immune resistance. Cancer Cell.

[49] Wu N., Veillette A. (2016). SLAM family receptors in normal immunity and immune pathologies. Curr. Opin. Immunol..

[50] Su S., Zhao J., Xing Y., Zhang X., Liu J., Ouyang Q., Chen J., Su F., Liu Q., Song E. (2018). Immune Checkpoint Inhibition Overcomes ADCP-Induced Immunosuppression by Macrophages. Cell.

[51] Vachon E., Martin R., Plumb J., Kwok V., Vandivier R.W., Glogauer M., Kapus A., Wang X., Chow C.W., Grinstein S. (2006). CD44 is a phagocytic receptor. Blood.

[52] May R.C., Caron E., Hall A., Machesky L.M. (2000). Involvement of the Arp2/3 complex in phagocytosis mediated by FcγR or CR3. Nat. Cell Biol..

[53] Olazabal I.M., Caron E., May R.C., Schilling K., Knecht D.A., Machesky L.M. (2002). Rho-kinase and myosin-II control phagocytic cup formation during CR, but not FcγR, phagocytosis. Curr. Biol..

[54] Zhang J., Guo J., Dzhagalov I., He Y.W. (2005). An essential function for the calcium-promoted Ras inactivator in Fcγ receptor-mediated phagocytosis. Nat. Immunol..

[55] Caron E., Hall A. (1998). Identification of two distinct mechanisms of phagocytosis controlled by different Rho GTPases. Science.

[56] Lorenzi R., Brickell P.M., Katz D.R., Kinnon C., Thrasher A.J. (2000). Wiskott-Aldrich syndrome protein is necessary for efficient IgG-mediated phagocytosis. Blood.

[57] D’Amico A.E., Wong A.C., Zajd C.M., Zhang X., Murali A., Trebak M., Lennartz M.R. (2021). PKC-epsilon regulates vesicle delivery and focal exocytosis for efficient IgG-mediated phagocytosis. J. Cell Sci..

[58] Leverrier Y., Okkenhaug K., Sawyer C., Bilancio A., Vanhaesebroeck B., Ridley A.J. (2003). Class I phosphoinositide 3-kinase p110beta is required for apoptotic cell and Fcγ receptor-mediated phagocytosis by macrophages. J. Biol. Chem..

[59] Suzuki T., Kono H., Hirose N., Okada M., Yamamoto T., Yamamoto K., Honda Z. (2000). Differential involvement of Src family kinases in Fcγ receptor-mediated phagocytosis. J. Immunol..

[60] Swanson J.A., Hoppe A.D. (2004). The coordination of signaling during Fc receptor-mediated phagocytosis. J. Leukoc. Biol..

[61] Marion S., Mazzolini J., Herit F., Bourdoncle P., Kambou-Pene N., Hailfinger S., Sachse M., Ruland J., Benmerah A., Echard A. (2012). The NF-kappaB signaling protein Bcl10 regulates actin dynamics by controlling AP1 and OCRL-bearing vesicles. Dev. Cell.

[62] Swanson J.A. (2008). Shaping cups into phagosomes and macropinosomes. Nat. Rev. Mol. Cell Biol..

[63] Braun V., Fraisier V., Raposo G., Hurbain I., Sibarita J.B., Chavrier P., Galli T., Niedergang F. (2004). TI-VAMP/VAMP7 is required for optimal phagocytosis of opsonised particles in macrophages. EMBO J..

[64] Niedergang F., Colucci-Guyon E., Dubois T., Raposo G., Chavrier P. (2003). ADP ribosylation factor 6 is activated and controls membrane delivery during phagocytosis in macrophages. J. Cell Biol..

[65] Nunes P., Demaurex N. (2010). The role of calcium signaling in phagocytosis. J. Leukoc. Biol..

[66] Peiser L., Mukhopadhyay S., Gordon S. (2002). Scavenger receptors in innate immunity. Curr. Opin. Immunol..

[67] Sulahian T.H., Imrich A., Deloid G., Winkler A.R., Kobzik L. (2008). Signaling pathways required for macrophage scavenger receptor-mediated phagocytosis: Analysis by scanning cytometry. Respir. Res..

[68] Strijbis K., Tafesse F.G., Fairn G.D., Witte M.D., Dougan S.K., Watson N., Spooner E., Esteban A., Vyas V.K., Fink G.R. (2013). Bruton’s Tyrosine Kinase (BTK) and Vav1 contribute to Dectin1-dependent phagocytosis of *Candida albicans* in macrophages. PLoS Pathog..

[69] Guilliams M., Bruhns P., Saeys Y., Hammad H., Lambrecht B.N. (2014). The function of Fcγ receptors in dendritic cells and macrophages. Nat. Rev. Immunol..

[70] Small A.G., Perveen K., Putty T., Patel N., Quinn P., Wechalekar M.D., Hii C.S., Quach A., Ferrante A. (2022). Neutrophils Require Activation to Express Functional Cell-Surface Complement Receptor Immunoglobulin. Front. Immunol..

[71] Sica A., Schioppa T., Mantovani A., Allavena P. (2006). Tumour-associated macrophages are a distinct M2 polarised population promoting tumour progression: Potential targets of anti-cancer therapy. Eur. J. Cancer.

[72] Musolino A., Gradishar W.J., Rugo H.S., Nordstrom J.L., Rock E.P., Arnaldez F., Pegram M.D. (2022). Role of Fcγ receptors in HER2-targeted breast cancer therapy. J. Immunother. Cancer.

[73] Shapouri-Moghaddam A., Mohammadian S., Vazini H., Taghadosi M., Esmaeili S.A., Mardani F., Seifi B., Mohammadi A., Afshari J.T., Sahebkar A. (2018). Macrophage plasticity, polarization, and function in health and disease. J. Cell Physiol..

[74] Kirschning C.J., Wesche H., Merrill Ayres T., Rothe M. (1998). Human toll-like receptor 2 confers responsiveness to bacterial lipopolysaccharide. J. Exp. Med..

[75] Toshchakov V., Jones B.W., Perera P.Y., Thomas K., Cody M.J., Zhang S., Williams B.R., Major J., Hamilton T.A., Fenton M.J. (2002). TLR4, but not TLR2, mediates IFN-beta-induced STAT1alpha/beta-dependent gene expression in macrophages. Nat. Immunol..

[76] Murakami Y., Tian L., Voss O.H., Margulies D.H., Krzewski K., Coligan J.E. (2014). CD300b regulates the phagocytosis of apoptotic cells via phosphatidylserine recognition. Cell Death Differ..

[77] Kong X.N., Yan H.X., Chen L., Dong L.W., Yang W., Liu Q., Yu L.X., Huang D.D., Liu S.Q., Liu H. (2007). LPS-induced down-regulation of signal regulatory protein alpha contributes to innate immune activation in macrophages. J. Exp. Med..

[78] Kong L., Ge B.X. (2008). MyD88-independent activation of a novel actin-Cdc42/Rac pathway is required for Toll-like receptor-stimulated phagocytosis. Cell Res..

[79] Pearson L.L., Castle B.E., Kehry M.R. (2001). CD40-mediated signaling in monocytic cells: Up-regulation of tumor necrosis factor receptor-associated factor mRNAs and activation of mitogen-activated protein kinase signaling pathways. Int. Immunol..

[80] Beatty G.L., Chiorean E.G., Fishman M.P., Saboury B., Teitelbaum U.R., Sun W., Huhn R.D., Song W., Li D., Sharp L.L. (2011). CD40 agonists alter tumor stroma and show efficacy against pancreatic carcinoma in mice and humans. Science.

[81] Liu M., O’Connor R.S., Trefely S., Graham K., Snyder N.W., Beatty G.L. (2019). Metabolic rewiring of macrophages by CpG potentiates clearance of cancer cells and overcomes tumor-expressed CD47-mediated ‘don’t-eat-me’ signal. Nat. Immunol..

[82] Hardie J., Mas-Rosario J.A., Ha S., Rizzo E.M., Farkas M.E. (2019). Macrophage activation by a substituted pyrimido[5,4-b]indole increases anti-cancer activity. Pharmacol. Res..

[83] Pyonteck S.M., Akkari L., Schuhmacher A.J., Bowman R.L., Sevenich L., Quail D.F., Olson O.C., Quick M.L., Huse J.T., Teijeiro V. (2013). CSF-1R inhibition alters macrophage polarization and blocks glioma progression. Nat. Med..

[84] Guerriero J.L., Sotayo A., Ponichtera H.E., Castrillon J.A., Pourzia A.L., Schad S., Johnson S.F., Carrasco R.D., Lazo S., Bronson R.T. (2017). Class IIa HDAC inhibition reduces breast tumours and metastases through anti-tumour macrophages. Nature.

[85] (2022). Mitochondrial fission in macrophages fuels phagocytosis of tumor cells. Nat. Cancer.

[86] Li J., Ye Y., Liu Z., Zhang G., Dai H., Li J., Zhou B., Li Y., Zhao Q., Huang J. (2022). Macrophage mitochondrial fission improves cancer cell phagocytosis induced by therapeutic antibodies and is impaired by glutamine competition. Nat. Cancer.

[87] Moradinasab S., Pourbagheri-Sigaroodi A., Ghaffari S.H., Bashash D. (2022). Targeting macrophage-mediated tumor cell phagocytosis: An overview of phagocytosis checkpoints blockade, nanomedicine intervention, and engineered CAR-macrophage therapy. Int. Immunopharmacol..

[88] Hossain M., Shim R., Lee W.Y., Sharpe A.H., Kubes P. (2022). Gata6^+^ resident peritoneal macrophages promote the growth of liver metastasis. Nat. Commun..

[89] Russ A., Hua A.B., Montfort W.R., Rahman B., Riaz I.B., Khalid M.U., Carew J.S., Nawrocki S.T., Persky D., Anwer F. (2018). Blocking “don’t eat me” signal of CD47-SIRPalpha in hematological malignancies, an in-depth review. Blood Rev..

[90] Hayat S.M.G., Bianconi V., Pirro M., Jaafari M.R., Hatamipour M., Sahebkar A. (2020). CD47: Role in the immune system and application to cancer therapy. Cell. Oncol..

[91] Brierley C.K., Staves J., Roberts C., Johnson H., Vyas P., Goodnough L.T., Murphy M.F. (2019). The effects of monoclonal anti-CD47 on RBCs, compatibility testing, and transfusion requirements in refractory acute myeloid leukemia. Transfusion.

[92] Sikic B.I., Lakhani N., Patnaik A., Shah S.A., Chandana S.R., Rasco D., Colevas A.D., O’Rourke T., Narayanan S., Papadopoulos K. (2019). First-in-Human, First-in-Class Phase I Trial of the Anti-CD47 Antibody Hu5F9-G4 in Patients With Advanced Cancers. J. Clin. Oncol..

[93] Advani R., Flinn I., Popplewell L., Forero A., Bartlett N.L., Ghosh N., Kline J., Roschewski M., LaCasce A., Collins G.P. (2018). CD47 Blockade by Hu5F9-G4 and Rituximab in Non-Hodgkin’s Lymphoma. N. Engl. J. Med..

[94] Gholamin S., Mitra S.S., Feroze A.H., Liu J., Kahn S.A., Zhang M., Esparza R., Richard C., Ramaswamy V., Remke M. (2017). Disrupting the CD47-SIRPalpha anti-phagocytic axis by a humanized anti-CD47 antibody is an efficacious treatment for malignant pediatric brain tumors. Sci. Transl. Med..

[95] Zeidan A.M., DeAngelo D.J., Palmer J., Seet C.S., Tallman M.S., Wei X., Raymon H., Sriraman P., Kopytek S., Bewersdorf J.P. (2022). Phase 1 study of anti-CD47 monoclonal antibody CC-90002 in patients with relapsed/refractory acute myeloid leukemia and high-risk myelodysplastic syndromes. Ann. Hematol..

[96] Yu X.Y., Qiu W.Y., Long F., Yang X.P., Zhang C., Xu L., Chang H.Y., Du P., Hou X.J., Yu Y.Z. (2018). A novel fully human anti-CD47 antibody as a potential therapy for human neoplasms with good safety. Biochimie.

[97] Ni H., Cao L., Wu Z., Wang L., Zhou S., Guo X., Gao Y., Jing H., Wu M., Liu Y. (2022). Combined strategies for effective cancer immunotherapy with a novel anti-CD47 monoclonal antibody. Cancer Immunol. Immunother..

[98] Peluso M.O., Adam A., Armet C.M., Zhang L., O’Connor R.W., Lee B.H., Lake A.C., Normant E., Chappel S.C., Hill J.A. (2020). The Fully human anti-CD47 antibody SRF231 exerts dual-mechanism antitumor activity via engagement of the activating receptor CD32a. J. Immunother. Cancer.

[99] Puro R.J., Bouchlaka M.N., Hiebsch R.R., Capoccia B.J., Donio M.J., Manning P.T., Frazier W.A., Karr R.W., Pereira D.S. (2020). Development of AO-176, a Next-Generation Humanized Anti-CD47 Antibody with Novel Anticancer Properties and Negligible Red Blood Cell Binding. Mol. Cancer Ther..

[100] Bouwstra R., van Meerten T., Bremer E. (2022). CD47-SIRPalpha blocking-based immunotherapy: Current and prospective therapeutic strategies. Clin. Transl. Med..

[101] Willingham S.B., Volkmer J.P., Gentles A.J., Sahoo D., Dalerba P., Mitra S.S., Wang J., Contreras-Trujillo H., Martin R., Cohen J.D. (2012). The CD47-signal regulatory protein alpha (SIRPa) interaction is a therapeutic target for human solid tumors. Proc. Natl. Acad. Sci. USA.

[102] Piccione E.C., Juarez S., Liu J., Tseng S., Ryan C.E., Narayanan C., Wang L., Weiskopf K., Majeti R. (2015). A bispecific antibody targeting CD47 and CD20 selectively binds and eliminates dual antigen expressing lymphoma cells. MAbs.

[103] Wang Y., Ni H., Zhou S., He K., Gao Y., Wu W., Wu M., Wu Z., Qiu X., Zhou Y. (2021). Tumor-selective blockade of CD47 signaling with a CD47/PD-L1 bispecific antibody for enhanced anti-tumor activity and limited toxicity. Cancer Immunol. Immunother..

[104] Chen Y.C., Shi W., Shi J.J., Lu J.J. (2022). Progress of CD47 immune checkpoint blockade agents in anticancer therapy: A hematotoxic perspective. J. Cancer Res. Clin. Oncol..

[105] Voets E., Parade M., Lutje Hulsik D., Spijkers S., Janssen W., Rens J., Reinieren-Beeren I., van den Tillaart G., van Duijnhoven S., Driessen L. (2019). Functional characterization of the selective pan-allele anti-SIRPalpha antibody ADU-1805 that blocks the SIRPalpha-CD47 innate immune checkpoint. J. Immunother. Cancer.

[106] Ansell S.M., Maris M.B., Lesokhin A.M., Chen R.W., Flinn I.W., Sawas A., Minden M.D., Villa D., Percival M.M., Advani A.S. (2021). Phase I Study of the CD47 Blocker TTI-621 in Patients with Relapsed or Refractory Hematologic Malignancies. Clin. Cancer Res..

[107] Querfeld C., Thompson J.A., Taylor M.H., DeSimone J.A., Zain J.M., Shustov A.R., Johns C., McCann S., Lin G.H.Y., Petrova P.S. (2021). Intralesional TTI-621, a novel biologic targeting the innate immune checkpoint CD47, in patients with relapsed or refractory mycosis fungoides or Sezary syndrome: A multicentre, phase 1 study. Lancet Haematol..

[108] Lakhani N.J., Chow L.Q.M., Gainor J.F., LoRusso P., Lee K.W., Chung H.C., Lee J., Bang Y.J., Hodi F.S., Kim W.S. (2021). Evorpacept alone and in combination with pembrolizumab or trastuzumab in patients with advanced solid tumours (ASPEN-01): A first-in-human, open-label, multicentre, phase 1 dose-escalation and dose-expansion study. Lancet Oncol..

[109] He H., Tu X., Zhang J., Acheampong D.O., Ding L., Ma Z., Ren X., Luo C., Chen Z., Wang T. (2015). A novel antibody targeting CD24 and hepatocellular carcinoma in vivo by near-infrared fluorescence imaging. Immunobiology.

[110] Sun F., Wang Y., Luo X., Ma Z., Xu Y., Zhang X., Lv T., Zhang Y., Wang M., Huang Z. (2019). Anti-CD24 Antibody-Nitric Oxide Conjugate Selectively and Potently Suppresses Hepatic Carcinoma. Cancer Res..

[111] Sousa L.G., Rajapakshe K., Rodriguez Canales J., Chin R.L., Feng L., Wang Q., Barrese T.Z., Massarelli E., William W., Johnson F.M. (2022). ISA101 and nivolumab for HPV-16^+^ cancer: Updated clinical efficacy and immune correlates of response. J. Immunother. Cancer.

[112] Armand P., Janssens A., Gritti G., Radford J., Timmerman J., Pinto A., Mercadal Vilchez S., Johnson P., Cunningham D., Leonard J.P. (2021). Efficacy and safety results from CheckMate 140, a phase 2 study of nivolumab for relapsed/refractory follicular lymphoma. Blood.

[113] Haag G.M., Springfeld C., Grun B., Apostolidis L., Zschabitz S., Dietrich M., Berger A.K., Weber T.F., Zoernig I., Schaaf M. (2022). Pembrolizumab and maraviroc in refractory mismatch repair proficient/microsatellite-stable metastatic colorectal cancer—The PICCASSO phase I trial. Eur. J. Cancer.

[114] Ni J., Zhou Y., Wu L., Ai X., Dong X., Chu Q., Han C., Wang X., Zhu Z. (2021). Sintilimab, stereotactic body radiotherapy and granulocyte-macrophage colony stimulating factor as second-line therapy for advanced non-small cell lung cancer: Safety run-in results of a multicenter, single-arm, phase II trial. Radiat. Oncol..

[115] Mantovani A., Allavena P., Marchesi F., Garlanda C. (2022). Macrophages as tools and targets in cancer therapy. Nat. Rev. Drug Discov..

[116] Wang Y., An E.K., Kim S.J., You S., Jin J.O. (2021). Intranasal Administration of Codium fragile Polysaccharide Elicits Anti-Cancer *Immunity* against Lewis Lung Carcinoma. Int. J. Mol. Sci..

[117] Dong H., Qi Y., Kong X., Wang Z., Fang Y., Wang J. (2022). PD-1/PD-L1 Inhibitor-Associated Myocarditis: Epidemiology, Characteristics, Diagnosis, Treatment, and Potential Mechanism. Front. Pharmacol..

[118] Qin W., Hu L., Zhang X., Jiang S., Li J., Zhang Z., Wang X. (2019). The Diverse Function of PD-1/PD-L Pathway Beyond Cancer. Front. Immunol..

[119] Su C., Wang H., Liu Y., Guo Q., Zhang L., Li J., Zhou W., Yan Y., Zhou X., Zhang J. (2020). Adverse Effects of Anti-PD-1/PD-L1 Therapy in Non-small Cell Lung Cancer. Front. Oncol..

[120] Hahn A.W., Gill D.M., Agarwal N., Maughan B.L. (2017). PD-1 checkpoint inhibition: Toxicities and management. Urol. Oncol..

[121] Sato R., Imamura K., Sakata S., Ikeda T., Horio Y., Iyama S., Akaike K., Hamada S., Jodai T., Nakashima K. (2019). Disorder of Coagulation-Fibrinolysis System: An Emerging Toxicity of Anti-PD-1/PD-L1 Monoclonal Antibodies. J. Clin. Med..

[122] Salik B., Smyth M.J., Nakamura K. (2020). Targeting immune checkpoints in hematological malignancies. J. Hematol. Oncol..

[123] Li W., Wang F., Guo R., Bian Z., Song Y. (2022). Targeting macrophages in hematological malignancies: Recent advances and future directions. J. Hematol. Oncol..

[124] Yang Y., Yang Z., Yang Y. (2021). Potential Role of CD47-Directed Bispecific Antibodies in Cancer Immunotherapy. Front. Immunol..

[125] Chauchet X., Cons L., Chatel L., Daubeuf B., Didelot G., Moine V., Chollet D., Malinge P., Pontini G., Masternak K. (2022). CD47xCD19 bispecific antibody triggers recruitment and activation of innate immune effector cells in a B-cell lymphoma xenograft model. Exp. Hematol. Oncol..

[126] Miller T.W., Amason J.D., Garcin E.D., Lamy L., Dranchak P.K., Macarthur R., Braisted J., Rubin J.S., Burgess T.L., Farrell C.L. (2019). Quantitative high-throughput screening assays for the discovery and development of SIRPalpha-CD47 interaction inhibitors. PLoS ONE.

[127] Wu Z., Weng L., Zhang T., Tian H., Fang L., Teng H., Zhang W., Gao J., Hao Y., Li Y. (2019). Identification of Glutaminyl Cyclase isoenzyme isoQC as a regulator of SIRPalpha-CD47 axis. Cell Res..

[128] Logtenberg M.E.W., Jansen J.H.M., Raaben M., Toebes M., Franke K., Brandsma A.M., Matlung H.L., Fauster A., Gomez-Eerland R., Bakker N.A.M. (2019). Glutaminyl cyclase is an enzymatic modifier of the CD47- SIRPalpha axis and a target for cancer immunotherapy. Nat. Med..

[129] Li Z., Gu X., Rao D., Lu M., Wen J., Chen X., Wang H., Cui X., Tang W., Xu S. (2021). Luteolin promotes macrophage-mediated phagocytosis by inhibiting CD47 pyroglutamation. Transl. Oncol..

[130] Kanemitsu N., Kiyonaga F., Mizukami K., Maeno K., Nishikubo T., Yoshida H., Ito H. (2021). Chronic treatment with the (iso-)glutaminyl cyclase inhibitor PQ529 is a novel and effective approach for glomerulonephritis in chronic kidney disease. Naunyn Schmiedeberg’s Arch. Pharmcol..

[131] Li C., Liu Y., Li D., Wang Q., Zhou S., Zhang H., Wang Y., He Z., Liu H., Sun J. (2022). Promising alternatives of CD47 monoclonal antibody: An injectable degradable hydrogel loaded with PQ912 for postoperative immunotherapy effectively blocks CD47-SIRPalpha signal. Theranostics.

[132] Lau J.L., Dunn M.K. (2018). Therapeutic peptides: Historical perspectives, current development trends, and future directions. Bioorg. Med. Chem..

[133] Wang H., Sun Y., Zhou X., Chen C., Jiao L., Li W., Gou S., Li Y., Du J., Chen G. (2020). CD47/SIRPalpha blocking peptide identification and synergistic effect with irradiation for cancer immunotherapy. J. Immunother. Cancer.

[134] Reganon E., Vila V., Aznar J., Garrido G., Estelles A., Berenguer J. (1987). Study of the formation of fibrin clot in cirrhotic patients. An approach to study of acquired dysfibrinogenemia. Thromb. Res..

[135] Moghimi S.M., Hunter A.C., Murray J.C. (2005). Nanomedicine: Current status and future prospects. FASEB J..

[136] Lian S., Xie X., Lu Y., Jia L. (2019). Checkpoint CD47 Function On Tumor Metastasis And Immune Therapy. Onco. Targets Ther..

[137] Steeland S., Vandenbroucke R.E., Libert C. (2016). Nanobodies as therapeutics: Big opportunities for small antibodies. Drug Discov. Today.

[138] Kijanka M., Dorresteijn B., Oliveira S., van Bergen en Henegouwen P.M. (2015). Nanobody-based cancer therapy of solid tumors. Nanomedicine.

[139] Ma L., Zhu M., Gai J., Li G., Chang Q., Qiao P., Cao L., Chen W., Zhang S., Wan Y. (2020). Preclinical development of a novel CD47 nanobody with less toxicity and enhanced anti-cancer therapeutic potential. J. Nanobiotechnol..

[140] Noble G.T., Stefanick J.F., Ashley J.D., Kiziltepe T., Bilgicer B. (2014). Ligand-targeted liposome design: Challenges and fundamental considerations. Trends Biotechnol..

[141] Cheng L., Zhang X., Tang J., Lv Q., Liu J. (2021). Gene-engineered exosomes-thermosensitive liposomes hybrid nanovesicles by the blockade of CD47 signal for combined photothermal therapy and cancer immunotherapy. Biomaterials.

[142] Ovais M., Guo M., Chen C. (2019). Tailoring Nanomaterials for Targeting Tumor-Associated Macrophages. Adv. Mater..

[143] Ramesh A., Kumar S., Nguyen A., Brouillard A., Kulkarni A. (2020). Lipid-based phagocytosis nanoenhancer for macrophage immunotherapy. Nanoscale.

[144] Kalluri R., LeBleu V.S. (2020). The biology, function, and biomedical applications of exosomes. Science.

[145] Nie W., Wu G., Zhang J., Huang L.L., Ding J., Jiang A., Zhang Y., Liu Y., Li J., Pu K. (2020). Responsive Exosome Nano-bioconjugates for Synergistic Cancer Therapy. Angew. Chem. Int. Ed. Engl..

[146] Kooijmans S.A.A., Vader P., Schiffelers R.M. (2017). Tumour-bound RNA-laden exosomes. Nat. Biomed. Eng..

[147] Zhang K.L., Wang Y.J., Sun J., Zhou J., Xing C., Huang G., Li J., Yang H. (2019). Artificial chimeric exosomes for anti-phagocytosis and targeted cancer therapy. Chem. Sci..

[148] Belhadj Z., He B., Deng H., Song S., Zhang H., Wang X., Dai W., Zhang Q. (2020). A combined “eat me/don’t eat me” strategy based on extracellular vesicles for anticancer nanomedicine. J. Extracell. Vesicles.

[149] Pham L.M., Poudel K., Phung C.D., Nguyen T.T., Pandit M., Nguyen H.T., Chang J.H., Jin S.G., Jeong J.H., Ku S.K. (2021). Preparation and evaluation of dabrafenib-loaded, CD47-conjugated human serum albumin-based nanoconstructs for chemoimmunomodulation. Colloids Surf. B Biointerfaces.

[150] Chen H., Cong X., Wu C., Wu X., Wang J., Mao K., Li J., Zhu G., Liu F., Meng X. (2020). Intratumoral delivery of CCL25 enhances immunotherapy against triple-negative breast cancer by recruiting CCR9^+^ T cells. Sci. Adv..

[151] Guo Z., Liu Y., Zhou H., Zheng K., Wang D., Jia M., Xu P., Ma K., Cui C., Wang L. (2019). CD47-targeted bismuth selenide nanoparticles actualize improved photothermal therapy by increasing macrophage phagocytosis of cancer cells. Colloids Surf. B Biointerfaces.

[152] Chen Q., Wang C., Zhang X., Chen G., Hu Q., Li H., Wang J., Wen D., Zhang Y., Lu Y. (2019). In situ sprayed bioresponsive immunotherapeutic gel for post-surgical cancer treatment. Nat. Nanotechnol..

[153] Zhang Y.R., Luo J.Q., Zhang J.Y., Miao W.M., Wu J.S., Huang H., Tong Q.S., Shen S., Leong K.W., Du J.Z. (2020). Nanoparticle-Enabled Dual Modulation of Phagocytic Signals to Improve Macrophage-Mediated Cancer Immunotherapy. Small.

[154] Liu X., Zheng J., Sun W., Zhao X., Li Y., Gong N., Wang Y., Ma X., Zhang T., Zhao L.Y. (2019). Ferrimagnetic Vortex Nanoring-Mediated Mild Magnetic Hyperthermia Imparts Potent Immunological Effect for Treating Cancer Metastasis. ACS Nano.

[155] Liu X., Yan B., Li Y., Ma X., Jiao W., Shi K., Zhang T., Chen S., He Y., Liang X.J. (2020). Graphene Oxide-Grafted Magnetic Nanorings Mediated Magnetothermodynamic Therapy Favoring Reactive Oxygen Species-Related Immune Response for Enhanced Antitumor Efficacy. ACS Nano.

[156] Kimura Y., Aoki H., Soyama T., Sakuragi A., Otsuka Y., Nomoto A., Yano S., Nishie H., Kataoka H., Aoyama M. (2022). Photodynamic therapy using mannose-conjugated chlorin e6 increases cell surface calreticulin in cancer cells and promotes macrophage phagocytosis. Med. Oncol..

[157] Liu Y., Wang J., Zhang J., Marbach S., Xu W., Zhu L. (2020). Targeting Tumor-Associated Macrophages by MMP2-Sensitive Apoptotic Body-Mimicking Nanoparticles. ACS Appl. Mater Interfaces.

[158] Zhou X., Liu X., Huang L. (2021). Macrophage-Mediated Tumor Cell Phagocytosis: Opportunity for Nanomedicine Intervention. Adv. Funct. Mater..

[159] Chen T.F., Li K.K., Zhu E.F., Opel C.F., Kauke M.J., Kim H., Atolia E., Wittrup K.D. (2018). Artificial Anti-Tumor Opsonizing Proteins with Fibronectin Scaffolds Engineered for Specificity to Each of the Murine FcγR Types. J. Mol. Biol..

[160] Cao X., Chen J., Li B., Dang J., Zhang W., Zhong X., Wang C., Raoof M., Sun Z., Yu J. (2022). Promoting antibody-dependent cellular phagocytosis for effective macrophage-based cancer immunotherapy. Sci. Adv..

[161] Kurdi A.T., Glavey S.V., Bezman N.A., Jhatakia A., Guerriero J.L., Manier S., Moschetta M., Mishima Y., Roccaro A., Detappe A. (2018). Antibody-Dependent Cellular Phagocytosis by Macrophages is a Novel Mechanism of Action of Elotuzumab. Mol. Cancer Ther..

[162] Lonial S., Dimopoulos M., Palumbo A., White D., Grosicki S., Spicka I., Walter-Croneck A., Moreau P., Mateos M.V., Magen H. (2015). Elotuzumab Therapy for Relapsed or Refractory Multiple Myeloma. N. Engl. J. Med..

[163] Shi Y., Fan X., Deng H., Brezski R.J., Rycyzyn M., Jordan R.E., Strohl W.R., Zou Q., Zhang N., An Z. (2015). Trastuzumab triggers phagocytic killing of high HER2 cancer cells in vitro and in vivo by interaction with Fcγ receptors on macrophages. J. Immunol..

[164] Overdijk M.B., Verploegen S., Bogels M., van Egmond M., Lammerts van Bueren J.J., Mutis T., Groen R.W., Breij E., Martens A.C., Bleeker W.K. (2015). Antibody-mediated phagocytosis contributes to the anti-tumor activity of the therapeutic antibody daratumumab in lymphoma and multiple myeloma. MAbs.

[165] Naicker S.D., Feerick C.L., Lynch K., Swan D., McEllistrim C., Henderson R., Leonard N.A., Treacy O., Natoni A., Rigalou A. (2021). Cyclophosphamide alters the tumor cell secretome to potentiate the anti-myeloma activity of daratumumab through augmentation of macrophage-mediated antibody dependent cellular phagocytosis. Oncoimmunology.

[166] Roghanian A., Hu G., Fraser C., Singh M., Foxall R.B., Meyer M.J., Lees E., Huet H., Glennie M.J., Beers S.A. (2019). Cyclophosphamide Enhances Cancer Antibody Immunotherapy in the Resistant Bone Marrow Niche by Modulating Macrophage FcγR Expression. Cancer Immunol. Res..

[167] Upton R., Banuelos A., Feng D., Biswas T., Kao K., McKenna K., Willingham S., Ho P.Y., Rosental B., Tal M.C. (2021). Combining CD47 blockade with trastuzumab eliminates HER2-positive breast cancer cells and overcomes trastuzumab tolerance. Proc. Natl. Acad. Sci. USA.

[168] Gul N., van Egmond M. (2015). Antibody-Dependent Phagocytosis of Tumor Cells by Macrophages: A Potent Effector Mechanism of Monoclonal Antibody Therapy of Cancer. Cancer Res..

[169] Gogesch P., Dudek S., van Zandbergen G., Waibler Z., Anzaghe M. (2021). The Role of Fc Receptors on the Effectiveness of Therapeutic Monoclonal Antibodies. Int. J. Mol. Sci..

[170] Bolli E., Scherger M., Arnouk S.M., Pombo Antunes A.R., Strassburger D., Urschbach M., Stickdorn J., De Vlaminck K., Movahedi K., Rader H.J. (2021). Targeted Repolarization of Tumor-Associated Macrophages via Imidazoquinoline-Linked Nanobodies. Adv. Sci..

[171] Ramesh A., Kumar S., Nandi D., Kulkarni A. (2019). CSF1R- and SHP2-Inhibitor-Loaded Nanoparticles Enhance Cytotoxic Activity and Phagocytosis in Tumor-Associated Macrophages. Adv. Mater..

[172] Yunna C., Mengru H., Lei W., Weidong C. (2020). Macrophage M1/M2 polarization. Eur. J. Pharmacol..

[173] Akilesh H.M., Buechler M.B., Duggan J.M., Hahn W.O., Matta B., Sun X., Gessay G., Whalen E., Mason M., Presnell S.R. (2019). Chronic TLR7 and TLR9 signaling drives anemia via differentiation of specialized hemophagocytes. Science.

[174] Ni K., Luo T., Culbert A., Kaufmann M., Jiang X., Lin W. (2020). Nanoscale Metal-Organic Framework Co-delivers TLR-7 Agonists and Anti-CD47 Antibodies to Modulate Macrophages and Orchestrate Cancer Immunotherapy. J. Am. Chem. Soc..

[175] Li F., Zheng X., Wang X., Xu J., Zhang Q. (2021). Macrophage polarization synergizes with oxaliplatin in lung cancer immunotherapy via enhanced tumor cell phagocytosis. Transl. Oncol..

[176] Li H., Somiya M., Kuroda S. (2021). Enhancing antibody-dependent cellular phagocytosis by Re-education of tumor-associated macrophages with resiquimod-encapsulated liposomes. Biomaterials.

[177] Kruit W.H., Suciu S., Dreno B., Mortier L., Robert C., Chiarion-Sileni V., Maio M., Testori A., Dorval T., Grob J.J. (2013). Selection of immunostimulant AS15 for active immunization with MAGE-A3 protein: Results of a randomized phase II study of the European Organisation for Research and Treatment of Cancer Melanoma Group in Metastatic Melanoma. J. Clin. Oncol..

[178] McQuade J.L., Homsi J., Torres-Cabala C.A., Bassett R., Popuri R.M., James M.L., Vence L.M., Hwu W.J. (2018). A phase II trial of recombinant MAGE-A3 protein with immunostimulant AS15 in combination with high-dose Interleukin-2 (HDIL2) induction therapy in metastatic melanoma. BMC Cancer.

[179] Sabado R.L., Pavlick A., Gnjatic S., Cruz C.M., Vengco I., Hasan F., Spadaccia M., Darvishian F., Chiriboga L., Holman R.M. (2015). Resiquimod as an immunologic adjuvant for NY-ESO-1 protein vaccination in patients with high-risk melanoma. Cancer Immunol. Res..

[180] Mehrotra S., Britten C.D., Chin S., Garrett-Mayer E., Cloud C.A., Li M., Scurti G., Salem M.L., Nelson M.H., Thomas M.B. (2017). Vaccination with poly(IC:LC) and peptide-pulsed autologous dendritic cells in patients with pancreatic cancer. J. Hematol. Oncol..

[181] Shen X., Burguillos M.A., Osman A.M., Frijhoff J., Carrillo-Jimenez A., Kanatani S., Augsten M., Saidi D., Rodhe J., Kavanagh E. (2016). Glioma-induced inhibition of caspase-3 in microglia promotes a tumor-supportive phenotype. Nat. Immunol..

[182] Fang Y., He Y., Wu C., Zhang M., Gu Z., Zhang J., Liu E., Xu Q., Asrorov A.M., Huang Y. (2021). Magnetism-mediated targeting hyperthermia-immunotherapy in “cold” tumor with CSF1R inhibitor. Theranostics.

[183] Han Y., Sun J., Yang Y., Liu Y., Lou J., Pan H., Yao J., Han W. (2022). TMP195 Exerts Antitumor Effects on Colorectal Cancer by Promoting M1 Macrophages Polarization. Int. J. Biol. Sci..

[184] Yue Y., Li F., Li Y., Wang Y., Guo X., Cheng Z., Li N., Ma X., Nie G., Zhao X. (2021). Biomimetic Nanoparticles Carrying a Repolarization Agent of Tumor-Associated Macrophages for Remodeling of the Inflammatory Microenvironment Following Photothermal Therapy. ACS Nano.

[185] Pan K., Farrukh H., Chittepu V., Xu H., Pan C.X., Zhu Z. (2022). CAR race to cancer immunotherapy: From CAR T, CAR NK to CAR macrophage therapy. J. Exp. Clin. Cancer Res..

[186] Keshavarz A., Salehi A., Khosravi S., Shariati Y., Nasrabadi N., Kahrizi M.S., Maghsoodi S., Mardi A., Azizi R., Jamali S. (2022). Recent findings on chimeric antigen receptor (CAR)-engineered immune cell therapy in solid tumors and hematological malignancies. Stem. Cell Res. Ther..

[187] Kang M., Lee S.H., Kwon M., Byun J., Kim D., Kim C., Koo S., Kwon S.P., Moon S., Jung M. (2021). Nanocomplex-Mediated In Vivo Programming to Chimeric Antigen Receptor-M1 Macrophages for Cancer Therapy. Adv. Mater..

[188] Chen Y., Yu Z., Tan X., Jiang H., Xu Z., Fang Y., Han D., Hong W., Wei W., Tu J. (2021). CAR-macrophage: A new immunotherapy candidate against solid tumors. Biomed. Pharmacother..

[189] Wang S., Yang Y., Ma P., Zha Y., Zhang J., Lei A., Li N. (2022). CAR-macrophage: An extensive immune enhancer to fight cancer. EBioMedicine.

[190] Morrissey M.A., Williamson A.P., Steinbach A.M., Roberts E.W., Kern N., Headley M.B., Vale R.D. (2018). Chimeric antigen receptors that trigger phagocytosis. Elife.

[191] Klichinsky M., Ruella M., Shestova O., Lu X.M., Best A., Zeeman M., Schmierer M., Gabrusiewicz K., Anderson N.R., Petty N.E. (2020). Human chimeric antigen receptor macrophages for cancer immunotherapy. Nat. Biotechnol..

[192] Zhang L., Tian L., Dai X., Yu H., Wang J., Lei A., Zhu M., Xu J., Zhao W., Zhu Y. (2020). Pluripotent stem cell-derived CAR-macrophage cells with antigen-dependent anti-cancer cell functions. J. Hematol. Oncol..

[193] Franken L., Schiwon M., Kurts C. (2016). Macrophages: Sentinels and regulators of the immune system. Cell Microbiol..

[194] Kim J., Bae J.S. (2016). Tumor-Associated Macrophages and Neutrophils in Tumor Microenvironment. Mediators Inflamm..

[195] van der Heide D., Weiskirchen R., Bansal R. (2019). Therapeutic Targeting of Hepatic Macrophages for the Treatment of Liver Diseases. Front. Immunol..

[196] Santoni M., Massari F., Montironi R., Battelli N. (2021). Manipulating macrophage polarization in cancer patients: From nanoparticles to human chimeric antigen receptor macrophages. Biochim Biophys Acta Rev. Cancer.

[197] Kim H.S., Sun X., Lee J.H., Kim H.W., Fu X., Leong K.W. (2019). Advanced drug delivery systems and artificial skin grafts for skin wound healing. Adv. Drug Deliv. Rev..

[198] Stylianopoulos T., Munn L.L., Jain R.K. (2018). Reengineering the Physical Microenvironment of Tumors to Improve Drug Delivery and Efficacy: From Mathematical Modeling to Bench to Bedside. Trends Cancer.

[199] Huang Y., Kim B.Y.S., Chan C.K., Hahn S.M., Weissman I.L., Jiang W. (2018). Improving immune-vascular crosstalk for cancer immunotherapy. Nat. Rev. Immunol..

[200] Huang Y., Yuan J., Righi E., Kamoun W.S., Ancukiewicz M., Nezivar J., Santosuosso M., Martin J.D., Martin M.R., Vianello F. (2012). Vascular normalizing doses of antiangiogenic treatment reprogram the immunosuppressive tumor microenvironment and enhance immunotherapy. Proc. Natl. Acad. Sci. USA.

[201] Ye Z.W., Yu Z.L., Chen G., Jia J. (2023). Extracellular vesicles in tumor angiogenesis and resistance to anti-angiogenic therapy. Cancer Sci..

[202] Jing H., Yang L., Qi J., Qiu L., Fu C., Li J., Yang M., Qi M., Fan N., Ji J. (2022). Safety and efficacy of daratumumab in Chinese patients with relapsed or refractory multiple myeloma: A phase 1, dose-escalation study (MMY1003). Ann. Hematol..

[203] Mateos M.V., Nahi H., Legiec W., Grosicki S., Vorobyev V., Spicka I., Hungria V., Korenkova S., Bahlis N., Flogegard M. (2020). Subcutaneous versus intravenous daratumumab in patients with relapsed or refractory multiple myeloma (COLUMBA): A multicentre, open-label, non-inferiority, randomised, phase 3 trial. Lancet Haematol..

[204] San-Miguel J., Usmani S.Z., Mateos M.V., van de Donk N., Kaufman J.L., Moreau P., Oriol A., Plesner T., Benboubker L., Liu K. (2021). Subcutaneous daratumumab in patients with relapsed or refractory multiple myeloma: Part 2 of the open-label, multicenter, dose-escalation phase 1b study (PAVO). Haematologica.

[205] Facon T., Kumar S.K., Plesner T., Orlowski R.Z., Moreau P., Bahlis N., Basu S., Nahi H., Hulin C., Quach H. (2021). Daratumumab, lenalidomide, and dexamethasone versus lenalidomide and dexamethasone alone in newly diagnosed multiple myeloma (MAIA): Overall survival results from a randomised, open-label, phase 3 trial. Lancet Oncol..

[206] Chari A., Martinez-Lopez J., Mateos M.V., Blade J., Benboubker L., Oriol A., Arnulf B., Rodriguez-Otero P., Pineiro L., Jakubowiak A. (2019). Daratumumab plus carfilzomib and dexamethasone in patients with relapsed or refractory multiple myeloma. Blood.

[207] Mateos M.V., Cavo M., Blade J., Dimopoulos M.A., Suzuki K., Jakubowiak A., Knop S., Doyen C., Lucio P., Nagy Z. (2020). Overall survival with daratumumab, bortezomib, melphalan, and prednisone in newly diagnosed multiple myeloma (ALCYONE): A randomised, open-label, phase 3 trial. Lancet.

[208] Sonneveld P., Chanan-Khan A., Weisel K., Nooka A.K., Masszi T., Beksac M., Spicka I., Hungria V., Munder M., Mateos M.V. (2023). Overall Survival With Daratumumab, Bortezomib, and Dexamethasone in Previously Treated Multiple Myeloma (CASTOR): A Randomized, Open-Label, Phase III Trial. J. Clin. Oncol..

[209] Dimopoulos M.A., Oriol A., Nahi H., San-Miguel J., Bahlis N.J., Usmani S.Z., Rabin N., Orlowski R.Z., Suzuki K., Plesner T. (2023). Overall Survival With Daratumumab, Lenalidomide, and Dexamethasone in Previously Treated Multiple Myeloma (POLLUX): A Randomized, Open-Label, Phase III Trial. J. Clin. Oncol..

[210] Modi S., Jacot W., Yamashita T., Sohn J., Vidal M., Tokunaga E., Tsurutani J., Ueno N.T., Prat A., Chae Y.S. (2022). Trastuzumab Deruxtecan in Previously Treated HER2-Low Advanced Breast Cancer. N. Engl. J. Med..

[211] Li B.T., Smit E.F., Goto Y., Nakagawa K., Udagawa H., Mazieres J., Nagasaka M., Bazhenova L., Saltos A.N., Felip E. (2022). Trastuzumab Deruxtecan in HER2-Mutant Non-Small-Cell Lung Cancer. N. Engl. J. Med..

[212] Siena S., Di Bartolomeo M., Raghav K., Masuishi T., Loupakis F., Kawakami H., Yamaguchi K., Nishina T., Fakih M., Elez E. (2021). Trastuzumab deruxtecan (DS-8201) in patients with HER2-expressing metastatic colorectal cancer (DESTINY-CRC01): A multicentre, open-label, phase 2 trial. Lancet Oncol..

[213] Salazar L.G., Lu H., Reichow J.L., Childs J.S., Coveler A.L., Higgins D.M., Waisman J., Allison K.H., Dang Y., Disis M.L. (2017). Topical Imiquimod Plus Nab-paclitaxel for Breast Cancer Cutaneous Metastases: A Phase 2 Clinical Trial. JAMA Oncol..

[214] Donin N.M., Chamie K., Lenis A.T., Pantuck A.J., Reddy M., Kivlin D., Holldack J., Pozzi R., Hakim G., Karsh L.I. (2017). A phase 2 study of TMX-101, intravesical imiquimod, for the treatment of carcinoma in situ bladder cancer. Urol Oncol..

[215] Shayan G., Kansy B.A., Gibson S.P., Srivastava R.M., Bryan J.K., Bauman J.E., Ohr J., Kim S., Duvvuri U., Clump D.A. (2018). Phase Ib Study of Immune Biomarker Modulation with Neoadjuvant Cetuximab and TLR8 Stimulation in Head and Neck Cancer to Overcome Suppressive Myeloid Signals. Clin. Cancer Res..

[216] Cassier P.A., Italiano A., Gomez-Roca C., Le Tourneau C., Toulmonde M., D’Angelo S.P., Weber K., Loirat D., Jacob W., Jegg A.M. (2020). Long-term clinical activity, safety and patient-reported quality of life for emactuzumab-treated patients with diffuse-type tenosynovial giant-cell tumour. Eur. J. Cancer.

[217] Cassier P.A., Italiano A., Gomez-Roca C.A., Le Tourneau C., Toulmonde M., Cannarile M.A., Ries C., Brillouet A., Muller C., Jegg A.M. (2015). CSF1R inhibition with emactuzumab in locally advanced diffuse-type tenosynovial giant cell tumours of the soft tissue: A dose-escalation and dose-expansion phase 1 study. Lancet Oncol..

[218] Spierenburg G., Grimison P., Chevreau C., Stacchiotti S., Piperno-Neumann S., Le Cesne A., Ferraresi V., Italiano A., Duffaud F., Penel N. (2022). Long-term follow-up of nilotinib in patients with advanced tenosynovial giant cell tumours: Long-term follow-up of nilotinib in TGCT. Eur. J. Cancer.

[219] Falchook G.S., Peeters M., Rottey S., Dirix L.Y., Obermannova R., Cohen J.E., Perets R., Frommer R.S., Bauer T.M., Wang J.S. (2021). A phase 1a/1b trial of CSF-1R inhibitor LY3022855 in combination with durvalumab or tremelimumab in patients with advanced solid tumors. Investig. N. Drugs.

[220] Lee J.H., Chen T.W., Hsu C.H., Yen Y.H., Yang J.C., Cheng A.L., Sasaki S.I., Chiu L.L., Sugihara M., Ishizuka T. (2020). A phase I study of pexidartinib, a colony-stimulating factor 1 receptor inhibitor, in Asian patients with advanced solid tumors. Investig. N. Drugs.

[221] Zhou Y.B., Zhang Y.M., Huang H.H., Shen L.J., Han X.F., Hu X.B., Yu S.D., Gao A.H., Sheng L., Su M.B. (2022). Pharmacodynamic, pharmacokinetic, and phase 1a study of bisthianostat, a novel histone deacetylase inhibitor, for the treatment of relapsed or refractory multiple myeloma. Acta Pharmacol. Sin..

[222] Heath E.I., Weise A., Vaishampayan U., Danforth D., Ungerleider R.S., Urata Y. (2022). Phase Ia dose escalation study of OBP-801, a cyclic depsipeptide class I histone deacetylase inhibitor, in patients with advanced solid tumors. Investig. N. Drugs.

[223] Collier K.A., Valencia H., Newton H., Hade E.M., Sborov D.W., Cavaliere R., Poi M., Phelps M.A., Liva S.G., Coss C.C. (2021). A phase 1 trial of the histone deacetylase inhibitor AR-42 in patients with neurofibromatosis type 2-associated tumors and advanced solid malignancies. Cancer Chemother. Pharmacol..

[224] Sborov D.W., Canella A., Hade E.M., Mo X., Khountham S., Wang J., Ni W., Poi M., Coss C., Liu Z. (2017). A phase 1 trial of the HDAC inhibitor AR-42 in patients with multiple myeloma and T- and B-cell lymphomas. Leuk. Lymphoma.

[225] Tsimberidou A.M., Beer P.A., Cartwright C.A., Haymaker C., Vo H.H., Kiany S., Cecil A.R.L., Dow J., Haque K., Silva F.A. (2021). Preclinical Development and First-in-Human Study of KA2507, a Selective and Potent Inhibitor of Histone Deacetylase 6, for Patients with Refractory Solid Tumors. Clin. Cancer Res..

[226] Tambo Y., Hosomi Y., Sakai H., Nogami N., Atagi S., Sasaki Y., Kato T., Takahashi T., Seto T., Maemondo M. (2017). Phase I/II study of docetaxel combined with resminostat, an oral hydroxamic acid HDAC inhibitor, for advanced non-small cell lung cancer in patients previously treated with platinum-based chemotherapy. Investig. N. Drugs.

[227] Quinn D.I., Tsao-Wei D.D., Twardowski P., Aparicio A.M., Frankel P., Chatta G., Wright J.J., Groshen S.G., Khoo S., Lenz H.J. (2021). Phase II study of the histone deacetylase inhibitor vorinostat (Suberoylanilide Hydroxamic Acid; SAHA) in recurrent or metastatic transitional cell carcinoma of the urothelium-an NCI-CTEP sponsored: California Cancer Consortium trial, NCI 6879. Investig. N. Drugs.

[228] Batlevi C.L., Crump M., Andreadis C., Rizzieri D., Assouline S.E., Fox S., van der Jagt R.H.C., Copeland A., Potvin D., Chao R. (2017). A phase 2 study of mocetinostat, a histone deacetylase inhibitor, in relapsed or refractory lymphoma. Br. J. Haematol..

[229] Jo J.H., Jung D.E., Lee H.S., Park S.B., Chung M.J., Park J.Y., Bang S., Park S.W., Cho S., Song S.Y. (2022). A phase I/II study of ivaltinostat combined with gemcitabine and erlotinib in patients with untreated locally advanced or metastatic pancreatic adenocarcinoma. Int. J. Cancer.

