# Supplementary material for: Anti-Tumor Strategies by Harnessing the Phagocytosis of Macrophages
Source: Cancers (Basel). 2023 May 11;15(10):2717. doi: 10.3390/cancers15102717 (PMC10216167; doi:10.3390/cancers15102717)
Supplement: Supplementary file 1 [file cancers-15-02717-s001.zip › cancers-2364402-supplementary.pdf]

**Table S1**: Clinical trials of anti-tumor therapy harnessing macrophage mediated-phagocytosis.

| Drug | Phase (status) | Tumor type | Administr-ation route | Strategy | Combination partners | mOS (m) | mPFS (m) | ORR (%) | TRAEs | NCT identifier | Ref. |
|---|---|---|---|---|---|---|---|---|---|---|---|
| **CD47/SIRP$\alpha$ blockade** | | | | | | | | | | | |
| TTI-621 | I (terminated) | Relapsed or refractory hematologic malignancies | i.v. | Mono/ combined | Rituximab, nivolumab | NA | NA | 13 | Infusion-related reactions, thrombocytopenia, chills, fatigue | NCT02663518 | [106] |
| TTI-621 | I (terminated) | Mycosis fungoides, Sézary syndrome | i.v. | Mono/ combined | Pembrolizumab, pegylated interferon alpha-2a | NA | NA | NA | Chills, injection site pain, fatigue | NCT02890368 | [107] |
| Evorpacept | I (ongoing) | Solid tumors, lymphoma | i.v. | Mono/ combined | Pembrolizumab, trastuzumab | 5.2 | 1.8 | NA | Neutropenia, thrombocytopenia, pancreatitis, death | NCT03013218 | [108] |
| CC-90002 | I (terminated) | Myeloid leukemia, myelodysplastic syndromes | i.v. | Mono | - | NA | NA | NA | Diarrhea, thrombocytopenia, febrile neutropenia, aspartate aminotransferase increase | NCT02641002 | [95] |
| Hu5F9-G4 | I (completed) | Solid Tumors | i.v. | Mono | - | NA | NA | NA | Transient anemia, hemagglutination on peripheral blood smear, fatigue, | NCT02216409 | [92] |

| | | | | | | | | | | | |
|---|---|---|---|---|---|---|---|---|---|---|---|
| | | | | | | | | | headaches, fever, chills | | |
| Hu5F9-G4 | I/II (ongoing) | B-cell non-Hodgkin's lymphoma | i.v. | Combined | Rituximab | NA | NA | NA | Chills, headache, anemia, infusion-related reactions | NCT02953509 | [93] |
| **ADCP-potentiating agent** | | | | | | | | | | | |
| Daratumumab | I (completed) | Relapsed or refractory MM | i.v. | Mono | - | NA | 6.7 | NA | Anemia, leukopenia, neutropenia, lymphopenia, upper respiratory tract infection, thrombocytopenia, hypokalemia | NCT02852837 | [202] |
| Daratumumab | III (ongoing) | Relapsed or refractory MM | s.c./i.v. | Mono | - | NA | 5.6(s.c.) 6.1(i.v.) | 41(s.c.) 37(i.v.) | Anemia, neutropenia, thrombocytopenia | NCT03277105 | [203] |
| Daratumumab | I (ongoing) | Relapsed or refractory MM | s.c. | Mono | - | NA | 12 | 52 | Lymphopenia, arthralgia, back pain, thrombocytopenia, diarrhea, nasopharyngitis | NCT02519452 | [204] |
| Daratumumab | III (ongoing) | Newly diagnosed MM | i.v. | Combined | lenalidomide, dexamethasone | NR | NR | NA | Neutropenia, anemia, lymphopenia, pneumonia | NCT02252172 | [205] |
| Daratumumab | I (ongoing) | Relapsed or refractory MM | i.v. | Combined | Carfilzomib, dexamethasone | NR | NR | 84 | Thrombocytopenia, lymphopenia, anemia, neutropenia | NCT01998971 | [206] |

| | | | | | | | | | | | |
|---|---|---|---|---|---|---|---|---|---|---|---|
| Daratumumab | III (ongoing) | Newly diagnosed MM | i.v. | Combined | Bortezomib, melphalan, prednisone | NA | 36·4 | 90.90 | Upper respiratory tract infections, bronchitis, viral upper respiratory tract infections, cough, diarrhea | NCT02195479 | [207] |
| Daratumumab | III (ongoing) | Previously treated MM | i.v. | Combined | Bortezomib, dexamethasone | 49.6 | 16.7 | NA | Thrombocytopenia, anemia, neutropenia, lymphopenia, pneumonia | NCT02136134 | [208] |
| Daratumumab | III (ongoing) | Previously treated MM | i.v. | Combined | Lenalidomide, dexamethasone | 67.6 | 53.3 | NA | Neutropenia, anemia, pneumonia, thrombocytopenia, diarrhea | NCT02076009 | [209] |
| Trastuzumab | III (ongoing) | Previously treated HER2-low advanced breast cancer | i.v. | Mono | - | 23.4 | 9.9 | NA | Nausea, fatigue, alopecia | NCT03734029 | [210] |
| Trastuzumab | II (ongoing) | HER2-mutant NSCLC | i.v. | Mono | - | 17.8 | 8.2 | NA | Neutropenia, adjudicated drug-related interstitial lung disease | NCT03505710 | [211] |
| Trastuzumab | II (completed) | HER2-expressing metastatic colorectal cancer | i.v. | Mono | - | NA | NA | NA | Lymphocytopenia, neutropenia, fatigue, pneumonia | NCT03384940 | [212] |

| | | | | | | | | | | | |
|---|---|---|---|---|---|---|---|---|---|---|---|
| Elotuzumab | III (completed) | Refractory MM | i.v. | Mono | - | NA | 19.4 | 79 | Lymphocytopenia, neutropenia, fatigue, pneumonia | NCT01239797 | [162] |
| **TLR agonists** | | | | | | | | | | | |
| AS15 | I (unknown) | Metastatic melanoma | i.m. | Combined | MAGE-A3 | 33 | 11.6 | NA | Pain, redness, swelling, fever, fatigue, chills, nausea | NCT00086866 | [177] |
| Resiquimod | I (completed) | High-risk melanoma | s.c. | Combined | NY-ESO-1 protein | NA | NA | NA | Influenza-like symptoms, injection site reactions | NCT00821652 | [179] |
| Imiquimod | II (completed) | Breast cancer cutaneous metastases | Applied topically | Combined | Albumin bound paclitaxel | NA | NA | 72 | 92% were grades 1 and 2 | NCT00821964 | [213] |
| Imiquimod | II (completed) | Carcinoma in situ bladder cancer | intravesical instillation | Mono | - | NA | NA | NA | Micturition urgency, dysuria, fatigue, hematuria | NCT01731652 | [214] |
| Poly-ICLC | I (completed) | Pancreatic cancer | i.m. | Combined | Peptide-pulsed DC vaccines | 7.7 | NA | NA | Fatigue, fever, myalgia, chills, night sweats, flashes | NCT01410968 | [180] |
| Motolimod | I (terminated) | SCCHN | s.c. | Combined | Cetuximab | NA | NA | NA | Injection site reaction, rash acneiform, flu-like symptoms, fatigue, diarrhea | NCT02124850 | [215] |
| **CSF1R inhibitors** | | | | | | | | | | | |

| | | | | | | | | | | | |
|---|---|---|---|---|---|---|---|---|---|---|---|
| Emactuzumab | I (completed) | dTGCT | i.v. | Mono | - | NA | NA | 71 | Pruritus, asthenia, oedema | NCT01494688 | [216] |
| Emactuzumab | I (completed) | Locally advanced dTGCT | i.v. | Mono/ combined | Paclitaxel | NA | NA | NA | Facial oedema, asthenia, pruritus, periorbital oedema, lupus erythematosus, erythema, dermohypodermitis | NCT01494688 | [217] |
| Tasigna | II (completed) | Advanced TGCT | p.o. | Mono | - | NA | 77 | NA | NA | NCT01261429 | [218] |
| LY3022855 | I (completed) | Advanced solid tumors | i.v. | Combined | Durvalumab, tremelimumab | NA | 1.87 | 4.2 | Anemia, pyrexia, peritonitis bacterial | NCT02718911 | [219] |
| Pexidartinib | I (completed) | Advanced solid tumors | p.o. | Mono | - | NA | NA | 13 | Anemia, increased aspartate aminotransferase level, decreased lymphocyte count | NCT02734433 | [220] |
| **HDAC inhibitors** | | | | | | | | | | | |
| Bisthianostat | I (ongoing) | Relapsed or refractory MM | p.o. | Mono | - | NA | NA | NA | Hematological TRAEs | NCT03618602 | [221] |
| OBP-801 | I (ongoing) | Advanced solid tumors | i.v. | Mono | - | NA | NA | NR | Abdominal pain, anemia and fatigue | NCT02414516 | [222] |

| | | | | | | | | | | | |
|---|---|---|---|---|---|---|---|---|---|---|---|
| AR-42 | II (completed) | Neurofibromatosis type 2-associated tumors, advanced solid malignancies | p.o. | Mono | - | NA | 3.6 | NA | Cytopenias, fatigue, and nausea | NCT01129193 | [223] |
| AR-42 | I (completed) | MM, T- and B-cell lymphomas | p.o. | Mono | - | NA | NA | NA | Cytopenias | NCT01129193 | [224] |
| KA2507 | I (completed) | Refractory solid tumors | p.o. | Mono | - | NA | 1.8 | NA | Fatigue, decreased appetite, urinary tract infection | NCT03008018 | [225] |
| Resminostat | II/III (terminated) | Advanced NSCLC | p.o. | Combined | Docetaxel | 15.3 | 4.1 | NA | Leukopenia, febrile neutropenia, thrombocytopenia, anorexia | NCT00473889 | [226] |
| Vorinostat | II (terminated) | Recurrent or metastatic transitional cell carcinoma of the urothelium | p.o. | Mono | - | 3.2 | 1.1 | NA | Cytopenias, thrombocytopenic bleeding | NCT00363883 | [227] |
| Mocetinostat | II (completed) | Relapsed or refractory lymphoma | p.o. | Mono | - | 12.3 (DLBCL) NR (FL) | 2.1 (DLBCL) 3.7 (FL) | 18.9 (DLBCL) 11.5 (FL) | Fatigue, nausea, diarrhea | NCT00359086 | [228] |

| | | | | | | | | | | | |
|---|---|---|---|---|---|---|---|---|---|---|---|
| Ivaltinostat | I/II (ongoing) | Untreated locally advanced or metastatic Pancreatic adenocarcinoma | i.v. | Combined | Gemcitabine, erlotinib | 8.6 | 5.3 | 25 | Decreased platelet count, decreased appetite, rash | NCT02737228 | [229] |

Abbreviations: mOS, median overall survival; mPFS, median progression-free survival; ORR, overall response rate; TRAE, treatment-related adverse event; i.v., intravenous; NA, no assessment; MM, multiple myeloma; s.c., subcutaneous; NR, not reached; i.m., intramuscular; NSCLC, non-small-cell lung cancer; IFA, incomplete Freund's adjuvant; SCCHN, squamous cell carcinoma of the head and neck ; dt-GCT, diffuse-type tenosynovial giant cell tumor; TGCT, tenosynovial giant cell tumor; DLBCL, diffuse large B-cell lymphoma; FL, follicular lymphoma.
